# Ventilatory efficiency: Physiological modelling and mechanistic validation

**DOI:** 10.1113/JP290456

**Published:** 2026-06-17

**Authors:** Paulo T Muller, Beate Stubbe, Till Ittermann, Erlandson S Ferreira, J A Neder, Ralf Ewert

**Affiliations:** ^1^ Faculty of Medicine, Department of Pneumology Federal University of Mato Grosso do Sul Campo Grande Brazil; ^2^ Department of Internal Medicine B University Medicine B Greifswald Germany; ^3^ Mathematical Institute, Biostatistical Department Federal University of Mato Grosso do Sul Campo Grande Mato Grosso do Sul Brazil; ^4^ Respiratory Investigation Unit, Division of Respirology, Department of Medicine Queen's University Kingston Ontario Canada

**Keywords:** diffusing capacity, exercise physiology, reference values, semi‐logarithmic modelling, ventilatory efficiency

## Abstract

**Abstract:**

Traditional indices such as the ${\dot{V}}_{E}$–${\dot{V}}_{CO_{2}}$ slope describe ventilatory efficiency within the submaximal, near‐linear domain of exercise but underrepresent the nonlinear ventilatory behaviour emerging beyond the first ventilatory threshold (VT_1_). We applied a semi‐logarithmic model that linearizes the post‐VT_1_ response by relating CO_2_ output to log‐transformed ventilation, extracting an empirical slope (*b_emp*) and normalizing it to a theoretical upper limit of CO_2_ clearance anchored to predicted maximal voluntary ventilation (MVV_pred), yielding the bounded ventilatory efficiency index $\eta {\dot{V}}_{E}$. In 1150 rigorously screened healthy adults (52.4% women; median age 49 years), $\eta {\dot{V}}_{E}$ exhibited minimal sex‐related variation (14.3% *vs*. 14.7%) and small positive associations with age (β = +0.058 ± 0.007, *P* < 0.0001) and FEV_1__pred (%) (β = +0.032 ± 0.008, *P* < 0.0001), accounting for ∼8.5% of total variance (*R* ^2^ = 0.085). Both empirical (median 3.3 [2.7–4.1] L·logL^−^^1^) and theoretical reference slopes (23.1 [19.5–27.3] L logL^−^^1^) declined with age, whereas $\eta {\dot{V}}_{E}$ remained stable across the lifespan, as confirmed by deterministic simulations demonstrating proportional coupling between ventilatory performance and theoretical capacity. In a *post hoc* cohort of individuals without cardiopulmonary disease but with isolated diffusive disturbance, multivariable regression identified $\eta {\dot{V}}_{E}$ as the only significant independent predictor of reduced diffusing capacity (*P* = 0.016), while age, height, sex and MVV_pred were non‐significant (all *P* > 0.20), indicating physiological, rather than geometric, determinants. By referencing ventilatory performance to a theoretical limit of CO_2_ removal, $\eta {\dot{V}}_{E}$ provides a reproducible, scale‐independent descriptor that refines the physiological interpretation of ventilatory efficiency across health, ageing and contrasting ventilatory constraints.

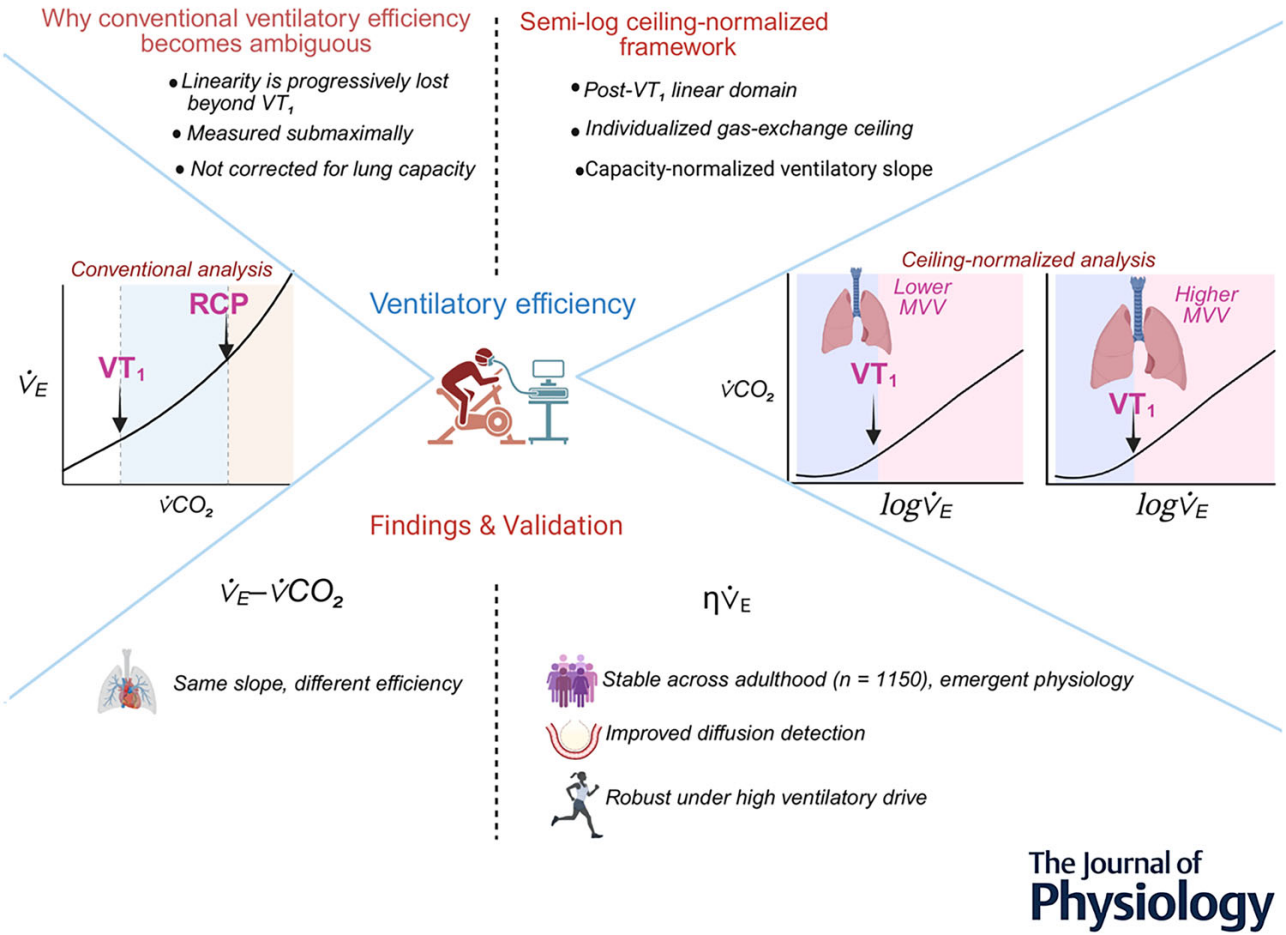

**Key points:**

- The ${\dot{V}}_{E}$–${\dot{V}}_{CO_{2}}$ slope and nadir underestimate key ventilatory adjustments during the most decisive phase of the exercise response – from the first ventilatory threshold (VT_1_) to peak exercise.
- This study introduces a semi‐logarithmic approach that linearizes the decisive post‐VT_1_ segment of the ventilatory response, better capturing its underlying physiological behaviour.
- The resulting slope, when normalized to a theoretical physiological limit for gas exchange and scaled to the predicted maximal voluntary ventilation, yields a bounded efficiency index ($\eta {\dot{V}}_{E}$, %).
- $\eta {\dot{V}}_{E}$ remained robustly stable and only weakly associated with age and lung function, while showing no meaningful dependence on sex or height in over 1000 healthy adults, from which valuable normative equations were derived.
- This framework integrates ventilatory drive, gas exchange and diffusion capacity, offering a unified and easily applicable tool for physiological and clinical evaluation of ventilatory efficiency.

## Graphical abstract

**Abstract figure legend** From post‐threshold ventilatory linearization to ceiling‐normalized efficiency – conceptual framework underlying $\eta {\dot{V}}_{E}$ Conventional ${\dot{V}}_{E}$–${\dot{V}}_{CO_{2}}$ slopes are typically derived under submaximal conditions and may become physiologically difficult to interpret beyond the first ventilatory threshold (VT_1_), where the original relationship progressively loses linearity. The proposed framework addresses this limitation by applying a semi‐logarithmic transformation (${\dot{V}}_{CO_{2}}$ *vs*. log_10_ ${\dot{V}}_{E}$) restricted to the linear post‐VT_1_ domain, yielding the empirical slope (b_emp). This slope is then referenced to an individualized theoretical gas‐exchange ceiling (b_ref), derived from predicted maximal voluntary ventilation (MVV_pred) and the theoretical upper limit of expired CO_2_ fraction. The resulting $\eta {\dot{V}}_{E}$ expresses ventilatory efficiency as a bounded physiological proportion of the individual's predicted ventilatory–gas‐exchange capacity.

## Introduction

Ventilatory efficiency during exercise is commonly inferred from the relationship between minute ventilation (${\dot{V}}_{E}$) and carbon dioxide output (${\dot{V}}_{CO_{2}}$), typically expressed as the ${\dot{V}}_{E}$–${\dot{V}}_{CO_{2}}$ slope or its nadir (Peterman et al., 2020; Phillips et al., 2020). Although widely used, these indices do not strictly quantify ventilatory efficiency *per se*, but rather reflect the proportionality between ventilatory drive and CO_2_ clearance under conditions of relative physiological stability (Neder et al., 2020; Ward, 2019; Ward, 2021). As such, their interpretability is largely confined to the submaximal domain, where acid–base balance is preserved and ventilatory reserve remains substantial (Muller & Saraiva, 2021; Nayor et al., 2020; Peterman et al., 2020).

This limitation becomes more evident beyond the first ventilatory threshold (VT_1_), where the ventilatory response progressively deviates from linearity and the slope loses direct physiological interpretability (Nayor et al., 2020; Phillips et al., 2020). In this domain, the combined influence of metabolic acidosis, chemoreflex stimulation, mechanical constraints and altered perfusion–diffusion matching leads to a nonlinear increase in ventilation relative to CO_2_ output (Phillips et al., 2020; Ramos et al., 2013; Whipp, 2007). Consequently, linear descriptors either underrepresent or distort the most physiologically relevant segment of the exercise response. This is reflected in contrasting clinical and physiological scenarios: individuals with impaired gas exchange and normal spirometry may exhibit apparently preserved ${\dot{V}}_{E}$–${\dot{V}}_{CO_{2}}$ slopes (Elbehairy et al., 2016; Muller et al., 2021), whereas highly trained individuals may display steeper slopes despite efficient ventilatory–metabolic coupling (Kasiak et al., 2024; Petek et al., 2022). Thus, current metrics may lead to misclassification when applied across heterogeneous physiological states.

A related conceptual limitation is the absence of a defined reference boundary against which ventilatory performance can be interpreted (Blake, 1991; Weibel et al., 1991). In the context of ventilatory–CO_2_ coupling, the observed ventilatory response reflects the interplay between neural drive and mechanical capacity, rather than a single underlying mechanism (Ward, 2021). As a result, similar proportional patterns may arise under fundamentally different physiological conditions, including states of augmented ventilatory drive (Arena et al., 2011; Petek et al., 2022) or, conversely, conditions in which mechanical constraints limit the achievable ventilatory response (Muller & Saraiva, 2021; Neder et al., 2015; Zuffo et al., 2024). In many biological systems, efficiency is inherently a bounded property, becoming physiologically meaningful only when expressed relative to a theoretical or functional limit (Bar‐Even et al., 2011; Zhu et al., 2010). For instance, mitochondrial oxidative phosphorylation operates consistently below its theoretical ATP yield, reflecting regulated efficiency within physiological constraints rather than maximal output (Salin et al., 2019). Without such a reference boundary, proportional changes in ventilatory performance cannot be distinguished from underlying physiological determinants, limiting interpretability (Alexander, 1981; Weibel et al., 1991).

To address these limitations, we developed a ventilatory efficiency index that explicitly targets two key constraints: the nonlinear behaviour of the ventilatory response beyond VT_1_ and the absence of a reference boundary for interpretation. By expressing ventilation on a logarithmic scale, the exponential rise in ${\dot{V}}_{E}$ beyond VT_1_ is transformed into an approximately linear relationship, allowing extraction of a slope that reflects the rate of CO_2_ clearance per order‐of‐magnitude increase in ventilatory drive (Muller, 2023). This empirical slope is then referenced to a theoretical framework based on predicted maximal ventilatory capacity (MVV_pred) and scaled to the upper theoretical limit of expired CO_2_ fraction, yielding a bounded efficiency index expressed as a percentage of a physiological ceiling, defined as a formal theoretical limit rather than an attainable physiological maximum (Muller, 2023; Muller & Saraiva, 2021).

This approach preserves proportionality across the full dynamic range of exercise and shifts interpretation from absolute ventilatory response to relative performance within physiological constraints. Importantly, it enables comparison across individuals differing in age, sex and body size by referencing ventilatory behaviour to an individualized theoretical capacity rather than to linear scaling alone (Muller, 2023).

However, anchoring the denominator to predicted ventilatory capacity raises the possibility that the resulting index could reflect structural factors, such as lung size, rather than true physiological efficiency. Therefore, the present study aimed to reinterpret and extend a ceiling‐referenced ventilatory efficiency framework derived from the ${\dot{V}}_{CO_{2}}$–log ${\dot{V}}_{E}$ relationship, establish normative values across a large population‐based cohort, and test its independence from spirometric scaling and demographic variables. We hypothesized that, when expressed relative to physiological potential, ventilatory efficiency would remain relatively stable across adulthood due to proportional coupling between performance and capacity. Within this framework, relative stability would reflect structural normalization, whereas deviations from this pattern would be expected to indicate disproportionate constraints on gas exchange or ventilatory control.

## Methods

### Ethical approval

All procedures conformed to the *Declaration of Helsinki* and were approved by the Ethics Committee of University Medicine Greifswald (approval no. BB 39/08). All participants provided written informed consent before inclusion. The present investigation represents a secondary, retrospective analysis of previously collected de‐identified data. The study conformed to the standards set by the *Declaration of Helsinki*, except for registration in a clinical‐trial database, as this was not an interventional clinical trial. Data collection and handling followed the governance framework of the Study of Health in Pomerania (SHIP), including standardized procedures for secure storage, controlled access and audited data transfer.

External construct validation was performed using two independent datasets comprising distinct physiological conditions (Barbosa et al., 2024; Muller et al., 2021). One cohort included smokers without airflow obstruction but with reduced lung diffusing capacity, representing early pulmonary microvascular impairment. The second cohort comprised endurance‐trained athletes performing both graded and supramaximal exercise, representing physiological extremes of ventilatory drive. These datasets were originally acquired under separate ethics approvals and analysed here using de‐identified data without additional participant contact. Importantly, no recalibration or retraining of the $\eta {\dot{V}}_{E}$ model was performed, ensuring independent external validation across heterogeneous physiological domains.

### Study design and population (SHIP)

This investigation used a population‐based cross‐sectional design with harmonized data from the SHIP. Standardized cardiopulmonary exercise testing (CPET) and spirometry were performed to establish sex‐ and age‐adjusted reference values for the new ventilatory efficiency index ($\eta {\dot{V}}_{E}$; pronounced *eta‐* ${\dot{V}}_{E}$). A rigorously screened sample of healthy adults was selected from SHIP cohorts after excluding smokers, individuals with cardiorespiratory or metabolic disease, and submaximal test efforts.

The study examined whether the theoretical reference slope – anchored to the theoretical limit of CO_2_ removal relative to ventilatory capacity – serves as a stable and unbiased denominator for $\eta {\dot{V}}_{E}$. Regression modelling, deterministic simulation and permutation testing were used to assess the stability and independence of this reference slope across sex, age and body size. Normative percentiles and predictive equations were then derived using quantile regression.

### Cardiopulmonary exercise testing

CPET was performed using a standardized ramp‐incremental, symptom‐limited protocol harmonized across all SHIP centres (Gläser et al., 2013). Tests were conducted in the upright seated position on calibrated electromagnetically braked cycle ergometers (Ergoselect 100/200; Ergoline GmbH, Germany) with breath‐by‐breath gas analysis (Oxycon Pro, VIASYS Healthcare, Germany) and Combitox masks.

The modified Jones protocol included 3 min of rest, 1 min of unloaded pedalling, a ramp increase of 16 W min^−^^1^, and 5 min of recovery. Respiratory variables – including ${\dot{V}}_{E}$, ${\dot{V}}_{CO_{2}}$ and ${\dot{V}}_{O_{2}}$ – were measured breath‐by‐breath and averaged in 5 s intervals. Flow sensors and gas analysers were calibrated daily using manufacturer‐recommended reference procedures and gases to ensure accuracy and reproducibility. During exercise testing, continuous ECG monitoring, intermittent non‐invasive blood pressure measurement, and pulse oximetry were performed. Peak values were defined as the highest 10 s averaged measurements obtained during maximal exertion or early recovery.

Tests were considered maximal if participants reached volitional exhaustion together with at least one of the following criteria: respiratory exchange ratio ≥1.10, attainment of ≥90% of age‐predicted maximal heart rate, or a plateau in ${\dot{V}}_{O_{2}}$. Only tests meeting predefined quality standards – based on technical adequacy, effort criteria and absence of premature termination – were included in the analyses.

### Dataset and eligibility criteria

This investigation used data from two harmonized population‐based cohorts of the SHIP (Völzke et al., 2022): SHIP‐TREND‐0 and SHIP‐START‐2. Together, these cohorts comprised 6753 participants aged 20–90 years, of whom 3067 underwent CPETs. Participants were recruited through stratified random sampling from population registries, ensuring representativeness across age, sex and residential distribution in northeastern Germany. For the present analyses, only individuals who completed both CPET and spirometry under standardized conditions were eligible for ventilatory efficiency assessment.

### Participant and exclusion criteria

To define a physiologically homogeneous reference group for ventilatory efficiency analyses, stringent exclusion criteria were applied based on medical history, medication use, spirometry, resting ECG and CPET performance. Participants were excluded if they were current smokers (*n* = 636), showed obstructive ventilatory patterns (FEV_1_/FVC < 70%; *n* = 221), or reported prior myocardial infarction (*n* = 78), cardiac surgery (*n* = 40) or chronic respiratory disease including self‐reported lung disease (*n* = 152), asthma (*n* = 95) or chronic bronchitis (*n* = 176). Individuals taking medications known to influence cardiovascular or respiratory physiology were also excluded: cardiac glycosides (ATC C01; *n* = 96), beta‐blockers (C07; *n* = 759), calcium channel blockers with diuretics (C08D; *n* = 23), and respiratory agents (R03‐class compounds; *n* = 74). Additional exclusions applied to participants with ischaemic ECG patterns (*n* = 694), bundle branch block (*n* = 97), pacemaker presence (*n* = 29) or reduced left‐ventricular fractional shortening (*n* = 1). Submaximal CPET effort (respiratory exchange ratio <1.10; *n* = 122) also led to exclusion, ensuring inclusion of only maximal or near‐maximal tests in the final dataset.

After accounting for overlapping criteria, 1917 individuals were excluded. The final analytic sample comprised 1150 rigorously screened participants, constituting the healthy reference population for establishing normative ventilatory efficiency parameters.

## Computational framework for $\eta {\dot{V}}_{E}$

### Extraction of the empirical slope (b_emp)

**Figure 1:**
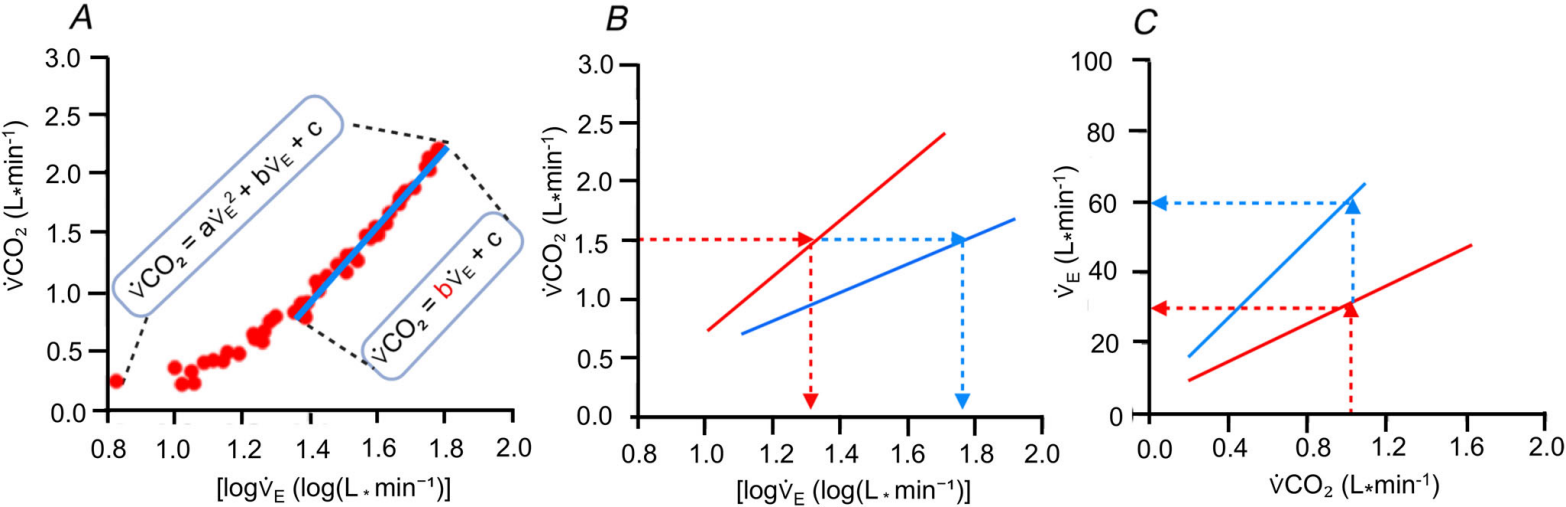
Semi‐log transformation of the ${\dot{V}}_{CO_{2}}$ – ${\dot{V}}_{E}$ relationship *A*, representative ${\dot{V}}_{CO_{2}}$–log ${\dot{V}}_{E}$ trajectory from a real test, illustrating the transition from a curvilinear to a linear segment. The slope of this linear portion (coefficient *b*) defines the empirical ${\dot{V}}_{CO_{2}}$–log ${\dot{V}}_{E}$ relationship used for analysis (blue line). *B*, interpretation of the semi‐logarithmic domain. The arrows quantify how much ventilation is required for a given CO_2_ output rather than labelling axes. To read the plot, fix a target ${\dot{V}}_{CO_{2}}$ (horizontal reference), follow it until it intersects the red (e.g. man) and blue (e.g. woman) regression lines, and project to the *x*‐axis to obtain log ${\dot{V}}_{E}$, then convert to ${\dot{V}}_{E}$ (${\dot{V}}_{E}$ = 10^log ${\dot{V}}_{E}$). For example, at ${\dot{V}}_{CO_{2}}$ ≈ 1.5 L min^−^^1^, the red line may intersect at log ${\dot{V}}_{E}$ ≈ 1.30 (${\dot{V}}_{E}$ ≈ 20 L min^−^^1^), whereas the blue line intersects at log ${\dot{V}}_{E}$ ≈ 1.75 (${\dot{V}}_{E}$ ≈ 56 L min^−^^1^). Thus, for the same CO_2_ output, the red slope entails less ventilation – indicating greater ‘ventilatory efficiency’ in the semi‐log domain. *C*, conventional linear ${\dot{V}}_{E}$–${\dot{V}}_{CO_{2}}$ representation. Here, the arrows again ask ‘how much ventilation for a given CO_2_?’ but the axes are inverted: ${\dot{V}}_{CO_{2}}$ is fixed on the *x*‐axis and ${\dot{V}}_{E}$ is read on the *y*‐axis at each intersection. For instance, at ${\dot{V}}_{CO_{2}}$ ≈ 1.0 L min^−^^1^, the red line yields ${\dot{V}}_{E}$ ≈ 35 L min^−^^1^, whereas the blue line gives ≈ 60 L min^−^^1^ – requiring greater ventilation and hence lower ‘efficiency’. These paired readings emphasize that interpretation in (*B*) is inverse to (*C*): a steeper slope in (*B*) implies higher ‘efficiency’ (less ${\dot{V}}_{E}$ for the same ${\dot{V}}_{CO_{2}}$), whereas a steeper slope in (*C*) implies lower ‘efficiency’ (more ${\dot{V}}_{E}$ for the same ${\dot{V}}_{CO_{2}}$). The term ‘efficiency’ in this paper is used comparatively, relative to the maximal theoretical slope (see *Efficiency Calculation* section).

**Figure 2:**
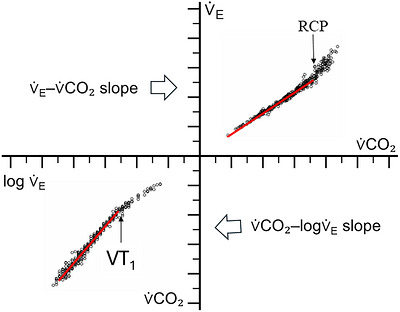
Geometric distinctions between the ${\dot{V}}_{E}$–${\dot{V}}_{CO_{2}}$ slope and the ${\dot{V}}_{CO_{2}}$–log ${\dot{V}}_{E}$ slope Upper right quadrant, conventional ${\dot{V}}_{E}$–${\dot{V}}_{CO_{2}}$ representation from a representative subject, showing the ventilatory response that becomes progressively curvilinear after the first ventilatory threshold (VT_1_) and approaches an exponential shape near the respiratory compensation point (RCP). Lower left quadrant, the same data with ventilation log‐transformed (${\dot{V}}_{CO_{2}}$–log ${\dot{V}}_{E}$ domain), yielding an approximately linear relationship from VT_1_ to peak exercise. The slope of this segment defines the empirical coefficient *b_emp*, used to derive the ventilatory efficiency index ($\eta {\dot{V}}_{E}$). Adapted from Muller, P.T. (2023), with permission.

The ventilatory efficiency index is defined as the ratio between the empirical ${\dot{V}}_{CO_{2}}$–log ${\dot{V}}_{E}$ slope (*b_emp*) and a theoretical reference slope (*b_ref* or ${\dot{V}}_{CO_{2}}$–log ${\dot{V}}_{E}$ slope max), expressed as a percentage (Muller, 2023). The empirical slope was obtained by plotting CO_2_ output (L min^−^^1^) against log_10_ ${\dot{V}}_{E}$ [log_10_(L min^−^^1^)] and performing linear regression on the linear segment beyond VT_1_, as illustrated in Fig. 1*A*. This segment represents the post‐VT_1_ linear response following the initial curvilinear transition. The resulting *b_emp* coefficient quantifies the increase in CO_2_ output associated with each one‐unit increase in log_10_ ${\dot{V}}_{E}$, corresponding to a tenfold increase in minute ventilation. This semi‐logarithmic transformation linearizes the post‐VT_1_ ventilatory response, enabling consistent slope estimation across the physiological range (Figs 1*B* and 2). The conceptual and graphical comparison between the conventional ${\dot{V}}_{E}$–${\dot{V}}_{CO_{2}}$ slope and the semi‐logarithmic approach is shown in Fig. 1*B* and *C*.

### Algorithmic *versus* physiological windowing

**Figure 3:**
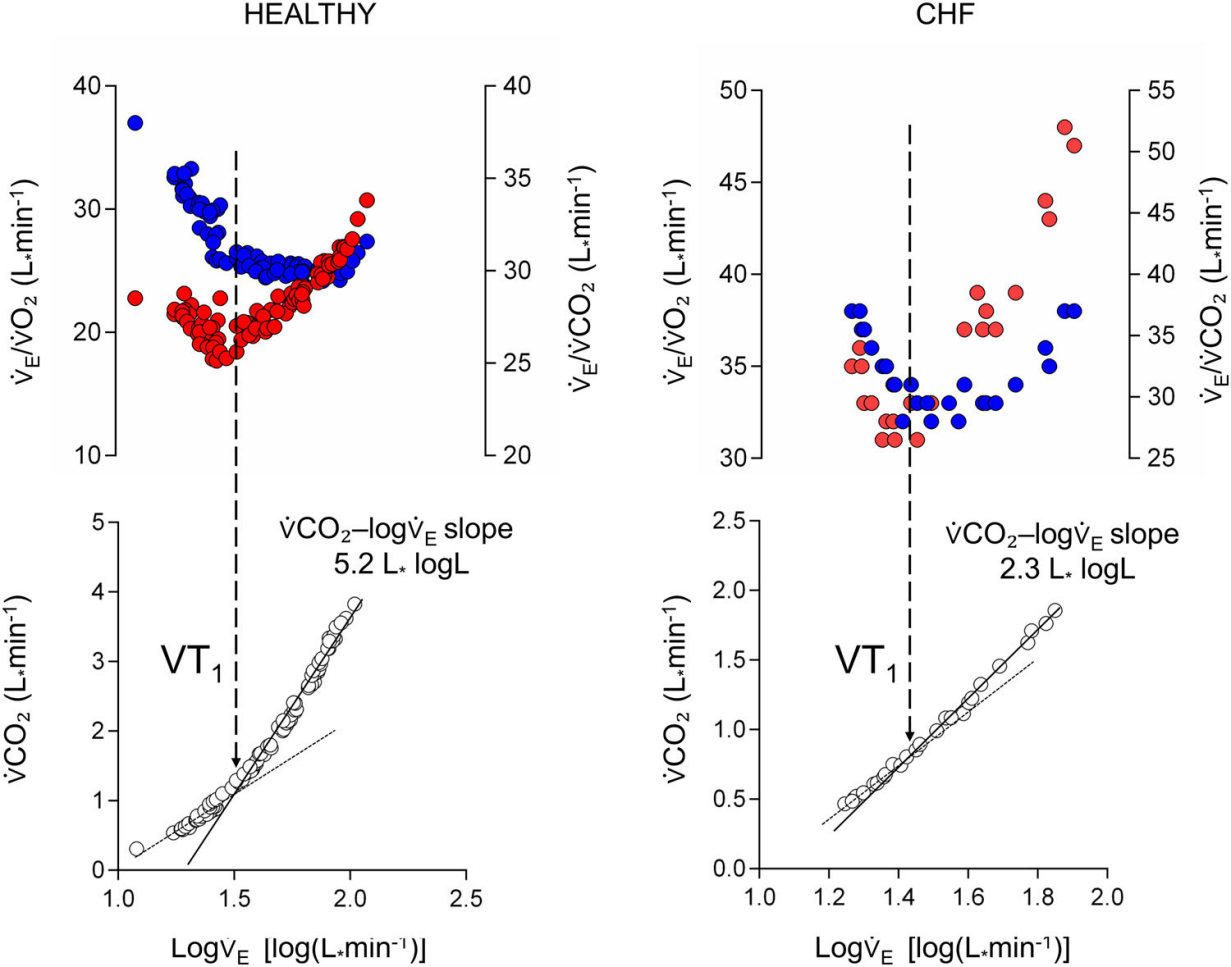
Temporally aligned ventilatory and metabolic responses Upper panels: ventilatory equivalents for carbon dioxide (${\dot{V}}_{E}$/${\dot{V}}_{CO_{2}}$) and oxygen (${\dot{V}}_{E}$/${\dot{V}}_{O_{2}}$) are plotted against the common *x*‐axis of log‐transformed minute ventilation (log ${\dot{V}}_{E}$). Of note, logarithmic transformation effectively linearizes relationships that follow power‐law or exponential behaviour (e.g. *y* = *a* × *x^b^* or *y* = *a* × e*^b^x*). However, because ${\dot{V}}_{E}$/${\dot{V}}_{CO_{2}}$ and ${\dot{V}}_{E}$/${\dot{V}}_{O_{2}}$ are composite ratios dependent on both ${\dot{V}}_{E}$ and ${\dot{V}}_{CO_{2}}$, applying the log transformation only to the common *x*‐axis partially cancels this effect, producing the residual curvature seen here. These patterns facilitate the identification of transition points corresponding to the first ventilatory threshold (VT_1_) and the respiratory compensation point. Lower panels: carbon dioxide output (${\dot{V}}_{CO_{2}}$, L min^−^^1^) plotted against the same log ${\dot{V}}_{E}$ axis for a healthy subject (left) and a patient with heart failure (right). The onset of the linear segment of the ${\dot{V}}_{CO_{2}}$–log ${\dot{V}}_{E}$ slope (solid black line) aligns closely with VT_1_, following an initial curvilinear (exponential) rise in ${\dot{V}}_{CO_{2}}$ (black dotted line). This transition coincides with the upward inflection in the ${\dot{V}}_{E}$/${\dot{V}}_{O_{2}}$ curve (red circles) and the onset of the isocapnic buffering phase, marked by a plateau in the ${\dot{V}}_{E}$/${\dot{V}}_{CO_{2}}$ ratio (blue circles). Thus, the vertical dashed line, marked by an arrow, denotes the approximate position of VT_1_. Adapted from Muller, P.T. (2023), with permission. CHF; Chronic Heart Failure

**Figure 4:**
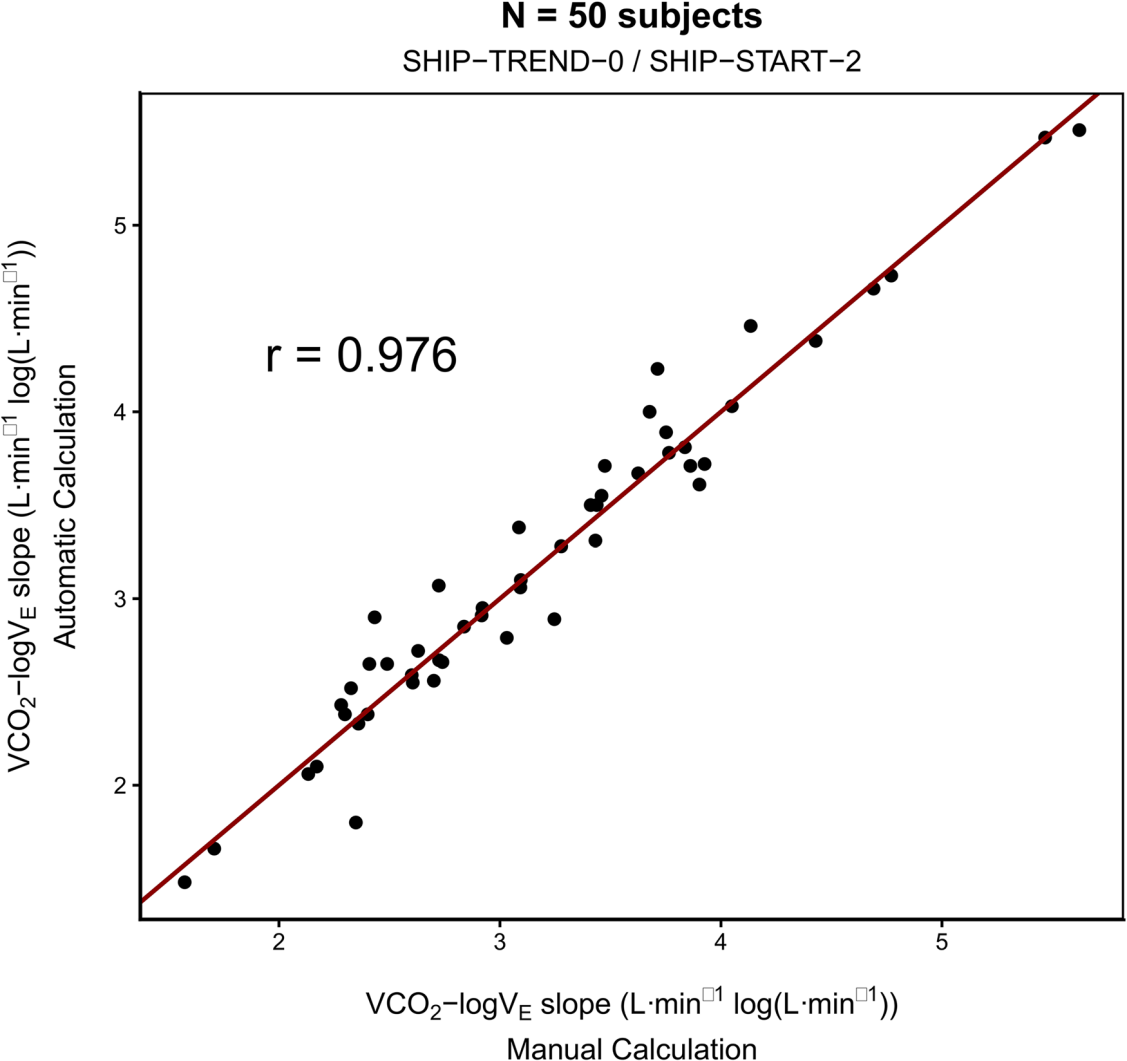
Correlation scatter plot between manual and automated calculations *N* = 50 individuals from SHIP cohorts.

The transition from the curvilinear to linear phase of the empirical ${\dot{V}}_{CO_{2}}$–log ${\dot{V}}_{E}$ relationship was initially identified using visual criteria (Muller, 2023) consistent with classical ventilatory threshold detection (Wasserman & Whipp, 1983; Whipp et al., 1982). To ensure reproducibility, an algorithmic approach was implemented in R statistical environment (R Foundation for Statistical Computing, Vienna, Austria; http://www.R‐project.org). A subset of 50 CPETs from the SHIP cohort was used for calibration and cross‐validation. Automated and manual slope estimates were independently derived (E.S.F. and P.T.M., respectively), with the automated method identifying the transition point by minimizing mean squared error between fitted segments. Manual estimation aligned the onset of linearity with VT_1_, confirmed by standard ventilatory equivalents (Fig. 3). Agreement between methods was high (*r* = 0.976), supporting the validity and reproducibility of automated *b_emp* estimation (Fig. 4).

### Theoretical ventilatory ceiling (*b_ref*)

**Figure 5:**
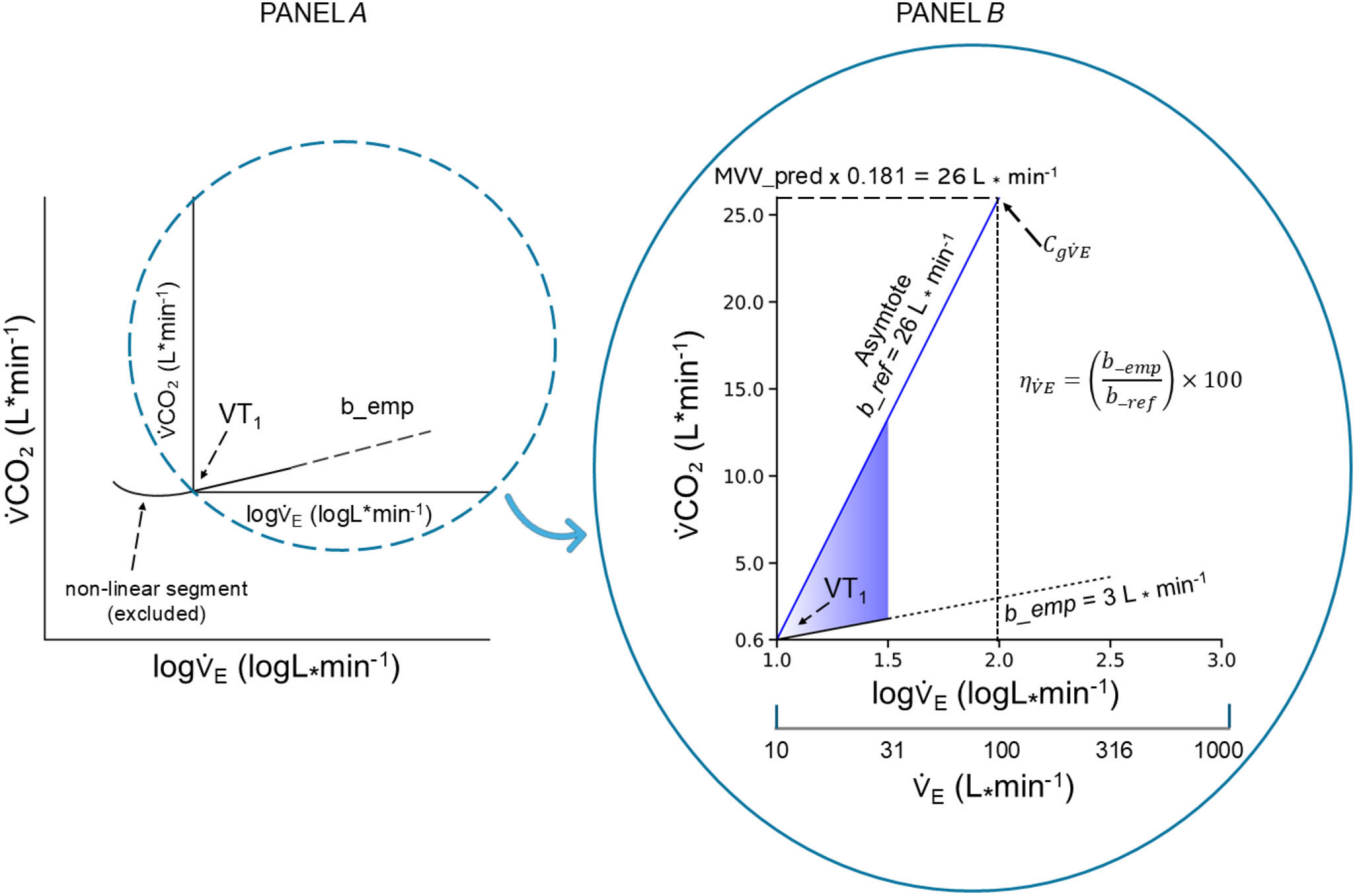
Semi‐logarithmic construction of ventilatory efficiency ($\eta {\dot{V}}_{E}$) *A*, the ${\dot{V}}_{CO_{2}}$–log ${\dot{V}}_{E}$ relationship. The initial curvilinear segment is excluded, and the empirical slope (*b_emp*) is fitted from VT_1_ onward, where the relationship becomes approximately linear. *B*, illustration of the derivation of $\eta {\dot{V}}_{E}$ by comparing the observed slope with a theoretical reference slope (*b_ref*). Both *b_emp* and *b_ref* are defined from the same origin (VT_1_ set to 0.0), such that the comparison depends only on slope, not intercept. In this example, post‐VT_1_ exercise spans log ${\dot{V}}_{E}$ = 1.0–1.5 (≈10–31.6 L min^−^^1^; blue shaded band). The dotted extension indicates that the empirical slope remains unchanged once the origin is fixed. The blue oblique line represents *b_ref*, derived from the ceiling constant C_($g{\dot{V}}_{E}$) = MVV_pred × 0.22 × 0.826. When Δlog ${\dot{V}}_{E}$ = 1.0, *b_ref* equals this constant, defining a reference slope corresponding to the theoretical maximal CO_2_ clearance per log‐unit increase in ventilation. A secondary *x*‐axis displays ventilation in L min^−^^1^ (antilog_10_ of log ${\dot{V}}_{E}$). Ventilatory efficiency is calculated as $\eta {\dot{V}}_{E}$ = (*b_emp* / *b_ref*) × 100. In this example, *b_emP* = 3.0 L min^−^^1^ and *b_reF* = 26 L min^−^^1^, yielding $\eta {\dot{V}}_{E}$ = 12%.

The purpose of *b_ref* is not to represent an attainable exercise value, but to provide an individualized upper‐bound reference against which the observed ventilatory response can be interpreted proportionally. Accordingly, *b_ref* defines a theoretical ventilatory–gas‐exchange ceiling based on MVV_pred scaled to an assumed upper limit for expired CO_2_ fraction (FECO_2__max ≈ 0.22) (Muller, 2023). This reference represents an idealized upper boundary for gas exchange and should be interpreted as a capacity‐based normalization framework rather than an achievable physiological maximum (Fig. 5).

Under typical physiological conditions, measured expired or end‐tidal CO_2_ fractions are substantially lower (≈0.04–0.06), emphasizing that FECO_2__max ≈ 0.22 represents a theoretical upper bound rather than an observed value. This limit is derived from alveolar gas constraints assuming near‐complete alveolar oxygen extraction and nitrogen as an inert background gas, and is therefore best understood as a formal reference condition. Additional details on the derivation and underlying assumptions are provided in Appendix A.

Importantly, this reference does not correspond to peak exercise ventilation, but to the upper ventilatory capacity of the respiratory system under standardized conditions, approximated by MVV_pred. Empirical data indicate that MVV typically exceeds peak exercise ventilation in more than 90% of individuals (Benítez‐Muñoz et al., 2025), supporting the interpretation of the ceiling as a capacity‐based construct rather than an exercise endpoint. In cardiopulmonary disease, peak ventilation remains well below predicted MVV, preserving a physiological margin between operational ventilation and the reference ceiling. This separation maintains interpretive resolution and minimizes the likelihood of ceiling saturation within the model. Additionally, ceiling saturation is unlikely because MVV_pred is scaled to an upper theoretical limit of gas exchange (FECO_2__max ≈ 0.22), a condition that is not physiologically attainable under normal conditions.

On this basis, a theoretical ceiling for ventilatory–gas‐exchange coupling can be expressed as:

(1) $$C\_\left(g{\dot{V}}_{E}\right)=\mathrm{MVV}\_\mathrm{pred}\times 0.22\times 0.826,$$

where C_($g{\dot{V}}_{E}$) represents the theoretical coupling coefficient integrating predicted maximal ventilation with the upper limit for gas exchange; MVV_pred denotes the estimated maximal voluntary ventilation, obtained from reference equations or approximated as FEV_1__pred × 41; 0.22 corresponds to the assumed maximal theoretical expired CO_2_ fraction; and 0.826 is the standard ATPS (ambient temperature and pressure, saturated)‐to‐STPD (standard temperature pressure, dry) conversion factor (*R*_mix). This formulation provides a stable reference for normalization of the empirical slope, anchoring ventilatory efficiency to a physiologically grounded upper boundary.

In the semi‐logarithmic domain (Δlog ${\dot{V}}_{E}$ = 1.0), *b_ref* corresponds to the geometric expression of this ceiling as a reference slope. Its apparent abstraction reflects the coordinate transformation rather than a lack of physiological grounding, and is consistent with other ceiling‐based constructs in physiology (Klabunde, 2022; Salin et al., 2019). In this formulation, *b_ref* represents the slope between a physiologically anchored point (VT_1_) and a projected upper‐bound constraint defined by C_($g{\dot{V}}_{E}$) within the transformed space, and is therefore uniquely determined by the same boundary conditions.

### Calculation of ventilatory efficiency ($\eta {\dot{V}}_{E}$)

In the present framework, ventilatory efficiency is expressed as the observed semi‐logarithmic CO_2_–ventilation slope relative to an individualized theoretical reference – a ceiling‐normalized property shaped by trade‐offs among gas exchange, mechanical cost, neural control and behavioural adaptability. This aligns with the physiological definition of efficiency as the ratio between achieved performance and its underlying capacity, typically expressed as a percentage (Blake, 1991), while recognizing that such values are inherently model‐dependent rather than governed by a universal energetic law. Operationally, the ventilatory efficiency index is defined as the ratio between the empirical and theoretical reference slopes, expressed as a percentage (Fig. 5):

(2) $$\eta_{\dot{V}E}=\left(\frac{b{\_}_{emp}}{b{\_}_{ref}}\right)\times 100,$$

where b_ref ≡ ∆(${\dot{V}}_{CO_{2}}$_ref) / ∆(log_10_ ${\dot{V}}_{E}$). For convenience of interpretation, when ∆(log_10_ ${\dot{V}}_{E}$) is set to 1.0 (i.e. one log‐unit, corresponding to a tenfold increase in ventilation), the geometric slope becomes numerically equivalent to the ceiling constant: b_re*f* = C_($g{\dot{V}}_{E}$) = MVV_pred × FECO_2__max × R_mix. This normalization does not constrain the analysis to this interval but provides a fixed reference scale for expressing the slope. Given that intercept terms cancel upon differentiation, making *b_ref* numerically equivalent to the reference gain constant (C_($g{\dot{V}}_{E}$)) – that is, the theoretical ${\dot{V}}_{CO_{2}}$_ref corresponding to one log‐unit of ventilation. The MVV_pred represents the predicted maximal voluntary ventilation, FECO_2__max denoting the theoretical physiological limit for expired CO_2_ fraction (≈ 0.22 under theoretical isothermal conditions with complete alveolar O_2_ uptake and nitrogen as the inert balance gas), and R_mix corresponding to the standard ATPS‐to‐STPD correction factor (≈ 0.826).

Although *b_ref* is an analytically derived theoretical constant rather than a fitted regression coefficient after intercept cancellation at VT_1_, it behaves as a stable upper boundary for the CO_2_ output rate within the model. Thus, it provides the essential reference formalism that anchors the *b_emp* to a theoretical ceiling, thereby allowing $\eta {\dot{V}}_{E}$ to be expressed as a dimensionless ratio. Despite the relatively complex theoretical framework that aligns all proposed limits and adjustments, the practical calculation of the new index is straightforward, with an algorithm box provided in Appendix B.

### Statistical modelling and multi‐tier validation

Age‐ and sex‐specific reference percentiles were derived using quantile regression (0.025, 0.5, 0.975), a distribution‐free approach robust to heteroscedasticity (Koenker et al., 2018). Models included age, body weight and height as candidate predictors, with variables retained at *P* < 0.10. Final equations were stratified by sex, and model performance was assessed using pseudo‐*R* ^2^ and residual diagnostics.

Validation combined deterministic simulation (Keener & Sneyd, 2009), regression modelling and external validation. A proof‐of‐concept simulation tested whether the observed age‐related patterns of $\eta {\dot{V}}_{E}$ could arise solely from the algebraic structure of its denominator. In this setting, both *b_emp*, derived from the semi‐logarithmic ${\dot{V}}_{CO_{2}}$–log ${\dot{V}}_{E}$ formulation used to define $\eta {\dot{V}}_{E}$, and *b_ref* were expressed as functions of predicted ventilatory capacity. Predicted FEV_1_ was obtained from regression equations derived from the SHIP cohort and used to estimate MVV_pred as 41 times FEV_1__pred (Koch et al., 2011). Three scenarios were examined: fixed *b_emp* with age‐varying MVV_pred, coupled age‐dependent variation of both terms, and age‐varying *b_emp* with fixed MVV_pred. All computations were implemented in R.

To assess construct independence, multiple linear regression models were fitted with $\eta {\dot{V}}_{E}$ as the dependent variable and age, sex and FEV_1__pred (%) as predictors in the SHIP reference sample (*n* = 1150):

(3) $$\eta {\dot{V}}_{E}=\beta_{0}+\beta_{1}\;\mathrm{Age}+\beta_{2}\;\mathrm{Sex}+\beta_{3}\mathrm{FEV}1\_\mathrm{pred}\;\left(\%\right)+\varepsilon .$$

Stability of regression estimates was further evaluated using permutation testing (10,000 iterations), relaxing distributional assumptions (Good, 2005). Model coefficients and *R* ^2^ were interpreted as indicators of construct association rather than predictive performance. Robustness analyses evaluated alternative MVV scaling (FEV_1__pred × 37, × 41, × 45) and FECO_2_, max (0.21–0.23), including joint variation of both parameters.

External validation was performed using an independent cohort of healthy smokers without airflow obstruction, incorporating diffusion capacity (DLco) (Muller et al., 2021). Logistic regression models were used to test associations between $\eta {\dot{V}}_{E}$ and DLco status (reduced *vs*. normal), adjusting for age, sex, height and FEV_1_. Discriminative performance was evaluated by the area under the receiver operating characteristic curve (AUC). The two groups were matched for age, sex, height and spirometric indices, allowing this analysis to specifically assess the relationship between $\eta {\dot{V}}_{E}$ and pulmonary gas‐exchange efficiency independently of morphometric and ventilatory factors. In addition, intra‐individual responsiveness of $\eta {\dot{V}}_{E}$ was evaluated in endurance‐trained athletes undergoing both graded and supramaximal exercise (Barbosa et al., 2024). Paired tests were used to compare $\eta {\dot{V}}_{E}$, ventilatory slopes and related gas‐exchange variables between exercise modes, thereby assessing the behaviour of the index under distinct dynamic ventilatory constraints.

## Results

### Normative behaviour of $\eta {\dot{V}}_{E}$ in healthy adults

The final sample comprised 1150 tests (52.4% women), with comparable age distributions between sexes. Median $\eta {\dot{V}}_{E}$ values were similar in women (14.3%) and men (14.7%). Although statistically significant (*P* = 0.0079), this difference was small and not physiologically meaningful.

**Figure 6:**
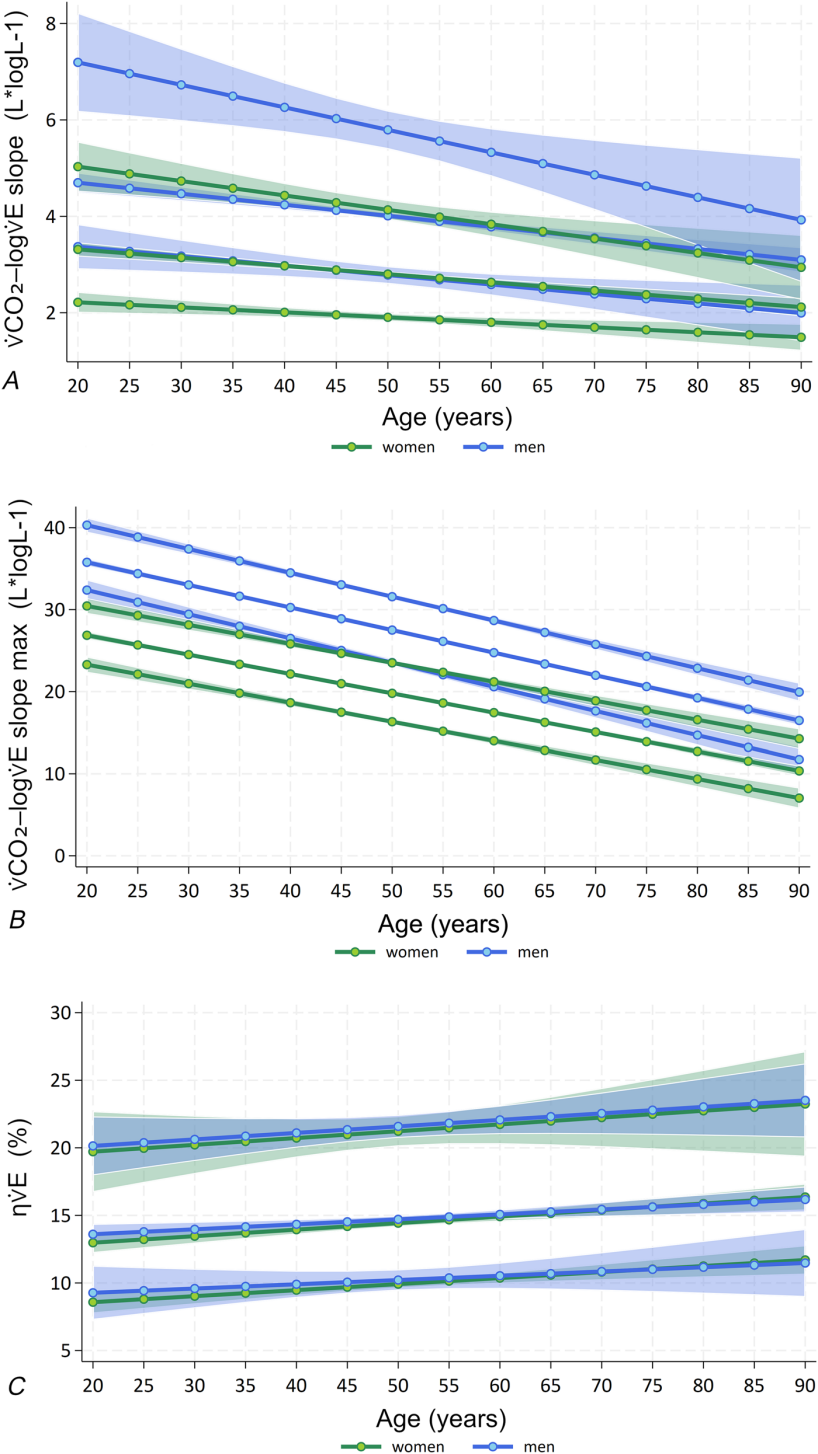
Age‐related behavior of $\eta {\dot{V}}_{E}$ components Quantile regression curves (5th, 50th and 95th percentiles) for the empirical ${\dot{V}}_{CO_{2}}$–log ${\dot{V}}_{E}$ slope (*A*), the theoretical reference slope (${\dot{V}}_{CO_{2}}$–log ${\dot{V}}_{E}$ slope max, *B*), and the ventilatory efficiency index $\eta {\dot{V}}_{E}$ (%) (*C*), stratified by sex. Shaded bands denote 95% confidence intervals. Both empirical and theoretical slopes decline progressively with age, whereas η ${\dot{V}}_{E}$ remains relatively stable across adulthood in men and women.

Reference characteristics are summarized in Table 1, with sex‐specific percentiles and predictive equations presented in Tables 2 and 3. Quantile regression analyses (Fig. 6) showed that both the empirical ${\dot{V}}_{CO_{2}}$–log ${\dot{V}}_{E}$ slope and the theoretical reference slope declined progressively with age, with consistently higher values in men. In contrast, $\eta {\dot{V}}_{E}$ remained relatively stable across the lifespan, with only a slight increase with age in both sexes. This pattern is consistent with proportional scaling between observed ventilatory performance and individualized theoretical capacity in health.

**Table 1:** Characteristics of the study population stratified by sex. Data are expressed as medians, 25th and 75th percentiles (*n* = 1150)

	Women	Men	Total
N	603 (52.4%)	547 (47.6%)	1150 (100.0%)
Age (years)	49 (40; 59)	50 (41; 60)	49 (41; 60)
Body height (cm)	165 (160; 169)	178 (173; 181)	171 (164; 178)
Body weight (kg)	69 (62; 78)	87 (78; 96)	78 (67; 90)
Body mass index (kg m^−2^)	25.7 (22.8; 29.0)	27.8 (25.3; 30.2)	26.7 (24.0; 29.5)
RER @ peak	1.20 (1.12; 1.28)	1.18 (1.10; 1.24)	1.19 (1.10; 1.26)
VO_2_ @ peak (mL min^−1^)	1628 (1397; 1873)	2587 (2236; 2991)	1986 (1592; 2574)
VEI ($\eta {\dot{V}}_{E}$,%)	14.3 (12.4; 16.5)	14.7 (12.9; 16.8)	14.5 (12.6; 16.6)
${\dot{V}}_{CO_{2}}$–log ${\dot{V}}_{E}$ slope	2.8 (2.4; 3.2)	4.0 (3.5; 4.6)	3.3 (2.7; 4.1)
${\dot{V}}_{CO_{2}}$–log ${\dot{V}}_{E}$ slope max	20.0 (17.3; 22.3)	27.6 (24.6; 30.2)	23.1 (19.5; 27.3)

Abbreviations: VEI: Ventilatory Efficiency Index; RER: Respiratory exchange ratio; VO_2_ @ peak: peak oxygen uptake; ${\dot{V}}_{CO_{2}}$–log ${\dot{V}}_{E}$ slope [L*logL^−1^]: empirical slope of CO_2_ output relative to log‐transformed minute ventilation; ${\dot{V}}_{CO_{2}}$–log ${\dot{V}}_{E}$ slope max [L*logL^−1^]: theoretically predicted maximal slope.

**Table 2:** Sex‐specific reference values expressed as 2.5th and 97.5th percentiles with 95% confidence interval (*n* = 1150)

	Women			Men		
	2.5th percentile	50th percentile	97.5th percentile	2.5th percentile	50th percentile	97.5th percentile
V. efficiency index ($\eta {\dot{V}}_{E}$, %)	9.6 (9.3; 9.9)	14.3 (13.9; 14.6)	22.3 (21.1; 23.5)	10.2 (9.5; 11.0)	14.7 (14.5; 15.0)	22.0 (21.1; 22.9)
${\dot{V}}_{CO_{2}}$–log ${\dot{V}}_{E}$ slope max (L*logL^−1^)	12.8 (12.0; 13.7)	20.0 (19.6; 20.5)	26.0 (25.3; 26.8)	19.1 (18.0; 20.1)	27.6 (27.0; 28.1)	35.0 (34.3; 35.7)
${\dot{V}}_{CO_{2}}$–log ${\dot{V}}_{E}$ slope (L*logL^−1^)	1.8 (1.7; 1.9)	2.8 (2.7; 2.9)	4.3 (4.1; 4.5)	2.6 (2.4; 2.8)	4.0 (3.9; 4.1)	6.1 (5.6; 6.6)

Abbreviations: ${\dot{V}}_{CO_{2}}$–log ${\dot{V}}_{E}$ slope: empirical slope of CO_2_ output relative to log‐transformed minute ventilation; ${\dot{V}}_{CO_{2}}$–log ${\dot{V}}_{E}$ slope max: theoretically predicted maximal slope.

**Table 3:** Final predictive equations for the three variables after modelling according to sex and 97.5th percentiles with 95% confidence interval (*n* = 1150)

	Formula	
	Women	Men
Ventilatory efficiency index ($\eta {\dot{V}}_{E}$_,_ %) 2.5th percentile median 97.5th percentile	24.7297 + 0.0446*age − 0.1035*height 41.0128 + 0.0483*age − 0.1646*height − 8806*(1/weight^2) 46.1157 + 0.0504*age − 0.1665*height	8.6428 + 0.0315*age 23.9220 + 0.0368*age − 0.0623*height 19.1797 + 0.04811*age
${\dot{V}}_{CO_{2}}$‐log ${\dot{V}}_{E}$ max (L*logL^−1^) 2.5th percentile median 97.5th percentile	−20.7214 − 0.1767*age + 0.2977*height −19.3751 − 0.1863*age + 0.2943*height −19.0543 − 0.1856*age + 0.2939*height	149.1819 − 0.2111*age − 1484*[1/sqrt(height)] −18.5593 − 0.2161*age + 0.3204*height −77.457 − 0.2156*age + 8.703*sqrt(height)
${\dot{V}}_{CO_{2}}$‐log ${\dot{V}}_{E}$ slope (L*logL^−1^) 2.5th percentile median 97.5th percentile	2.4228 − 0.0104*age 0.2361 − 0.0171*age + 0.0174*height + 0.0077*weight 5.6275 − 0.0299*age	3.7631 − 0.0196*age −0.2017 − 0.0229*age + 0.0302*height 8.1281 − 0.0467*age

Abbreviations: ${\dot{V}}_{CO_{2}}$–log ${\dot{V}}_{E}$ slope: empirical slope of CO_2_ output relative to log‐transformed minute ventilation; ${\dot{V}}_{CO_{2}}$–log ${\dot{V}}_{E}$ max: theoretically predicted maximal slope.

### Robustness of $\eta {\dot{V}}_{E}$ to alternative scaling assumptions

Sensitivity analyses were performed to evaluate the impact of alternative assumptions for ventilatory capacity (MVV_pred = FEV_1__pred × 37, × 41, × 45) and maximal expired CO_2_ fraction (FECO_2__max = 0.21–0.23), including joint variation of both parameters (Table 4). Absolute $\eta {\dot{V}}_{E}$ values varied proportionally across assumptions, with predictable shifts in distribution percentiles and regression slopes. However, the correlation between $\eta {\dot{V}}_{E}$ and FEV_1_ remained unchanged across all conditions (*r* = −0.176), and percentile ordering was preserved. These findings indicate that $\eta {\dot{V}}_{E}$ is sensitive to scaling in absolute terms but structurally robust in its relationship with lung function.

**Table 4:** Joint sensitivity analysis of $\eta {\dot{V}}_{E}$ across alternative assumptions for MVV scaling and FECO_2__max

MVV factor	FECO_2__max	Percentiles, $\eta {\dot{V}}_{E}$ (P10/P50/P90)	Slope (FEV_1_‐ $\eta {\dot{V}}_{E}$)	*r*
37	0.21	13.10/16.84/22.05	−0.891	−0.176
37	0.22	12.51/16.08/21.05	−0.850	−0.176
37	0.23	11.96/15.38/20.13	−0.813	−0.176
41	0.21	11.82/15.20/19.81	−0.804	−0.176
41	0.22	11.29/14.52/19.00	−0.767	−0.176
41	0.23	10.80/13.88/18.17	−0.734	−0.176
45	0.21	10.77/13.85/18.04	−0.732	−0.176
45	0.22	10.28/13.23/17.31	−0.699	−0.176
45	0.23	9.84/12.64/16.56	−0.669	−0.176

*Note*: Values are expressed as the 10th, 50th and 90th percentiles (P10/P50/P90) of $\eta {\dot{V}}_{E}$. Slope represents the linear regression coefficient for the relationship between FEV_1_ (L) and $\eta {\dot{V}}_{E}$. The *r* denotes the Pearson correlation coefficient. Absolute $\eta {\dot{V}}_{E}$ values varied proportionally across assumptions, whereas the correlation structure remained unchanged.

### Comparison with the ${\dot{V}}_{E}$–${\dot{V}}_{CO_{2}}$ slope

**Figure 7:**
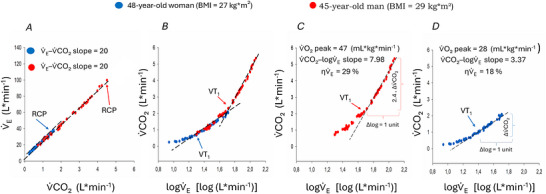
Comparative trajectories of the conventional ${\dot{V}}_{E}$–${\dot{V}}_{CO_{2}}$ slope and the semi‐logarithmic ${\dot{V}}_{CO_{2}}$–log ${\dot{V}}_{E}$ relationship during incremental exercise in two healthy, physically active, non‐athlete participants Closed blue circles denote data from a 48‐year‐old woman (BMI = 27 kg m^−^^2^), and closed red circles denote data from a 45‐year‐old man (BMI = 29 kg m^−^^2^). *A*, the characteristic linear ${\dot{V}}_{E}$–${\dot{V}}_{CO_{2}}$ slope, measured from exercise onset to the respiratory compensation point (RCP). *B*, the curvilinear (quadratic) relationship between ${\dot{V}}_{CO_{2}}$ and log ${\dot{V}}_{E}$, with linear regression applied to the segment beyond the first ventilatory threshold (VT_1_). *C* and *D*, the individual responses extracted from *B*, using identical axis scales to facilitate direct comparison. For an equivalent interval of Δlog ${\dot{V}}_{E}$ = 1 unit (a tenfold increase in ventilation), the male participant exhaled approximately 2.4 times more CO_2_ than the female participant, corresponding to roughly a 60% reduction in ventilatory requirement for the same CO_2_ removal and indicating substantially higher ‘ventilatory efficiency’ in the male subject. Importantly, this difference occurred despite both individuals exhibiting nearly identical ${\dot{V}}_{E}$–${\dot{V}}_{CO_{2}}$ slopes in *A*. The male participant also achieved a higher peak ${\dot{V}}_{O_{2}}$, and when referenced to maximal voluntary ventilation, his ventilatory efficiency index ($\eta {\dot{V}}_{E}$) reached 29%, reflecting the physiological significance of the semi‐logarithmic approach beyond VT_1_.

A comparative analysis between the conventional ${\dot{V}}_{E}$–${\dot{V}}_{CO_{2}}$ slope and the semi‐logarithmic ${\dot{V}}_{CO_{2}}$–log ${\dot{V}}_{E}$ framework is shown in Fig. 7. Despite nearly identical ${\dot{V}}_{E}$–${\dot{V}}_{CO_{2}}$ slopes in two representative individuals (Fig. 7*A*), marked differences emerged when ventilatory behaviour was analysed using the ${\dot{V}}_{CO_{2}}$–log ${\dot{V}}_{E}$ slope (Fig. 7*B*) and $\eta {\dot{V}}_{E}$ (Fig. 7*C* and Fig. 7*D*).

The semi‐logarithmic approach revealed substantial divergence in ventilatory efficiency beyond VT_1_, with higher *b_emp* values corresponding to lower ventilatory requirements for a given CO_2_ output. This contrast highlights the greater discriminatory capacity of the ${\dot{V}}_{CO_{2}}$–log ${\dot{V}}_{E}$ framework compared with conventional linear descriptors.

### Deterministic validation of $\eta {\dot{V}}_{E}$ stability

**Figure 8:**
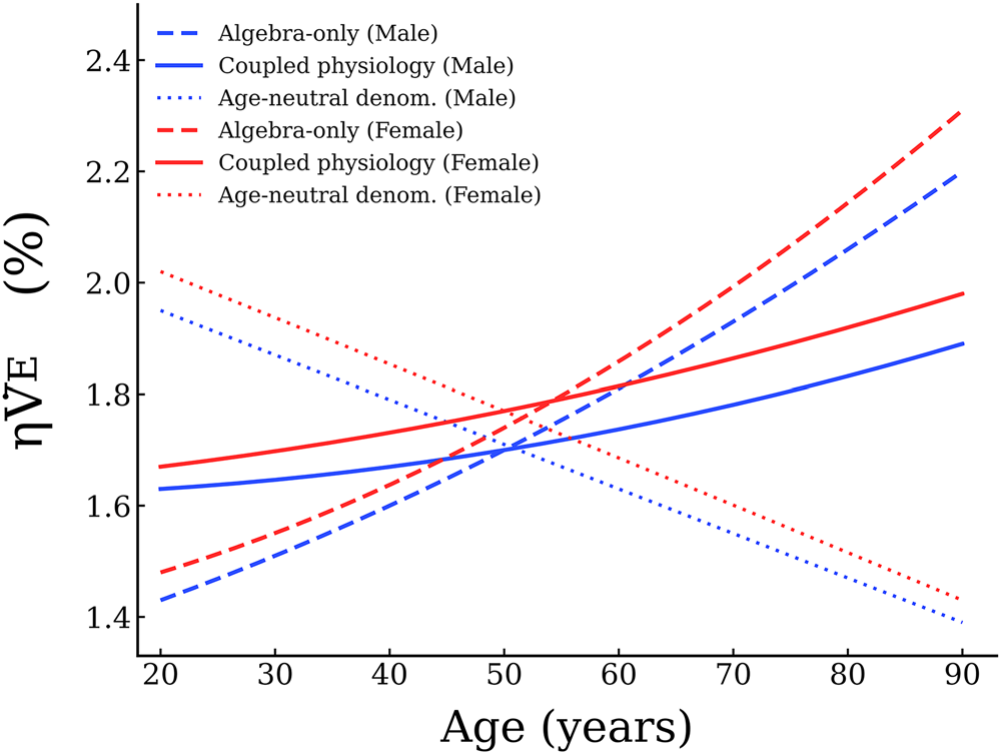
Deterministic validation illustrating that $\eta {\dot{V}}_{E}$ behaves as a ratio‐based, non‐algebraic variable, remaining stable across ageing as a result of proportional ventilatory–metabolic coupling The three simulated scenarios are shown: *Algebra‐only*, *Coupled physiology* and *Age‐neutral denominator*. In the *Algebra‐only* model, $\eta {\dot{V}}_{E}$ rises with age due solely to the declining denominator when the numerator is fixed. In the *Coupled physiology* condition, $\eta {\dot{V}}_{E}$ remains nearly age‐neutral because both *b*_*emp* and *MVV*_pred decrease proportionally, preserving their ratio. When age dependence is removed from the denominator (*Age‐neutral denominator*), $\eta {\dot{V}}_{E}$ decreases with age, demonstrating that the observed stability of $\eta {\dot{V}}_{E}$ reflects coordinated physiological covariation rather than an algebraic artefact. For simplicity, the constants (FECO_2_ and R_mix) were omitted from the denominator, leaving MVV_pred as the sole term.

Deterministic simulations (Fig. 8) showed that $\eta {\dot{V}}_{E}$ behaviour depends on the coupling between its empirical and structural components. When this coupling was preserved (coupled‐physiology model), $\eta {\dot{V}}_{E}$ remained nearly stable across age, with only minimal variation. In contrast, disruption of this relationship produced divergent trajectories, with $\eta {\dot{V}}_{E}$ increasing or decreasing depending on whether age dependence was confined to the denominator or removed from it. These findings indicate that the age stability of $\eta {\dot{V}}_{E}$ reflects coordinated physiological coupling rather than an algebraic artefact.

### Regression‐based construct validation

**Figure 9:**
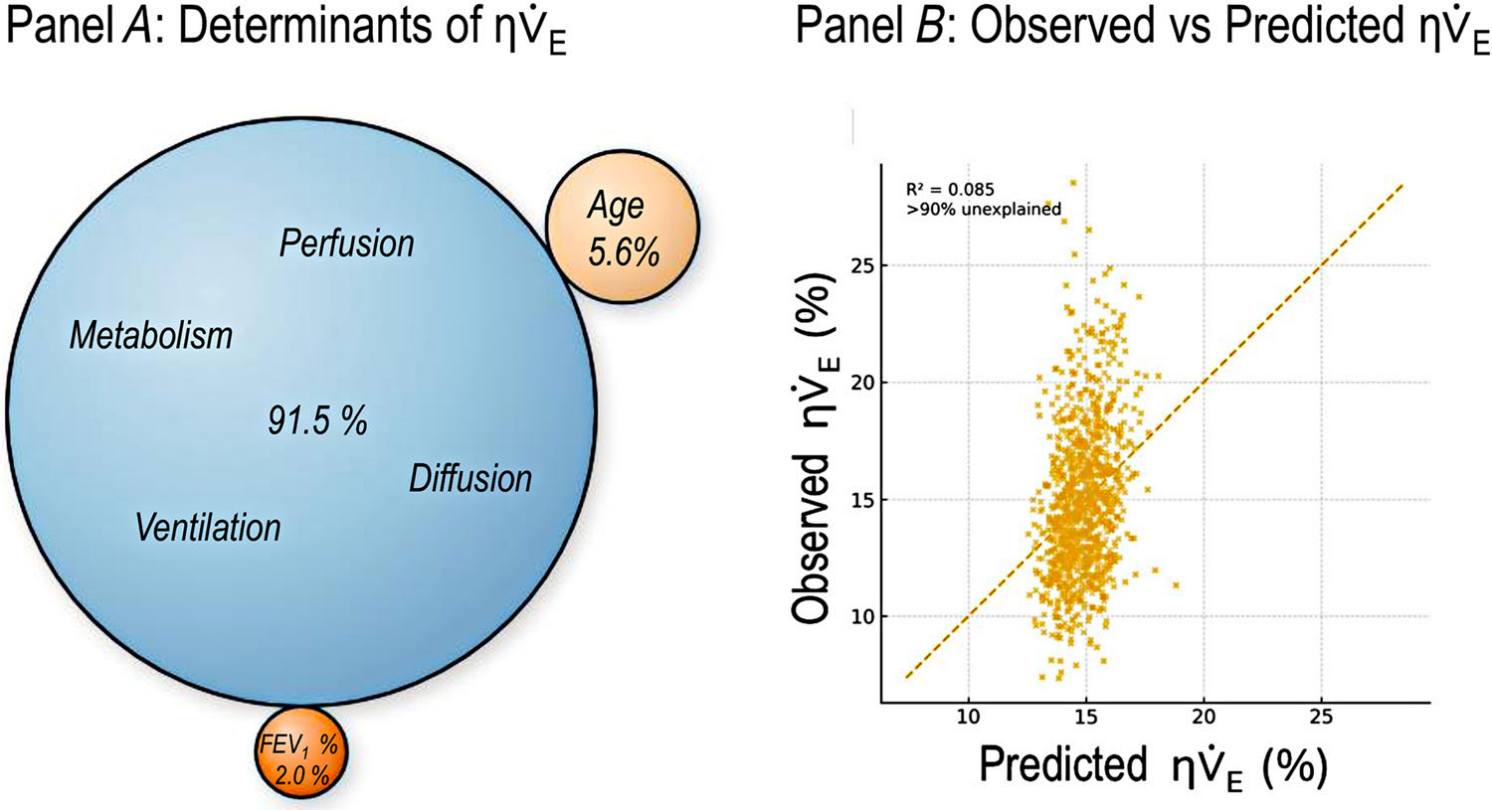
Determinants and variance explained in $\eta {\dot{V}}_{E}$ among healthy individuals *A*, relative contributions of physiological domains to $\eta {\dot{V}}_{E}$ variance. Structural factors such as FEV_1__pred (%) and age account for only modest shares of total variability, with most of the variance reflecting integrative physiological determinant. *B*, relationship between observed and predicted $\eta {\dot{V}}_{E}$ values from the multivariate regression model (*R* ^2^ = 0.085), illustrating that most of $\eta {\dot{V}}_{E}$ variability reflects physiological rather than structural factors.

Multiple linear regression analysis showed that $\eta {\dot{V}}_{E}$ was largely independent of conventional morphometric and spirometric predictors (Table 5). Age and FEV_1__pred (%) exhibited small but statistically significant positive associations (β = 0.058 and 0.032, respectively; *P* < 0.0001), whereas sex had no significant effect. The model explained a limited proportion of variance (*R* ^2^ = 0.085), indicating that most variability in $\eta {\dot{V}}_{E}$ is not accounted for by anthropometric or spirometric factors (Fig. 9). Residual analyses showed no evidence of systematic bias related to body size or spirometric scaling. Permutation testing (10,000 iterations) confirmed the robustness of these findings, with significant effects for age and FEV_1__pred and no effect of sex. All analyses are fully reproducible using the dataset and scripts available in public repositories (Zenodo and OSF; https://doi.org/10.5281/zenodo.17508654).

**Table 5:** Multiple regression of $\eta {\dot{V}}_{E}$ on age, sex and FEV_1__pred (%) (healthy SHIP cohort, *n* = 1150)

Term	Beta (OLS)	SE (OLS)	T (OLS)	*P‐*value (OLS)	Beta(OLS) (HC3)	SE(OLS/) (HC3)	t(OLS) (HC3)	p(OLS) (HC3)
Intercept	8.510	0.731	11.60	**<0.0001**	8.510	0.846	10.1	**<0.0001**
Age (years)	+0.058	0.006	8.35	**<0.0001**	0.058	0.007	8.07	**<0.0001**
M (*vs*. F)	+0.253	0.179	1.47	**0.142**	0.263	0.179	1.47	**0.141**
FEV_1_% pred	+0.032	0.006	4.93	**<0.0001**	0.032	0.008	4.04	**<0.0001**

*Note*: *Model fit statistics for the fitted regression model: *R* ^2^ **= 0.0868**, adjusted *R* ^2^ **= 0.0850**. F: 54.4 *P‐value* (model) < 0.0001.

Abbreviations: OLS = ordinary least squares; HC3 = heteroskedasticity‐consistent covariance estimator.

### External validation across ventilatory constraint domains

External validation was performed across two distinct physiological contexts, including diffusion‐limited gas exchange in smokers and high ventilatory drive in athletes.

### Diffusion‐limited gas exchange

**Figure 10:**
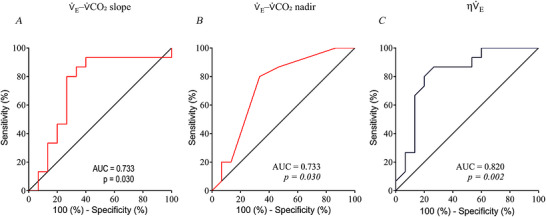
Proof‐of‐concept application of $\eta {\dot{V}}_{E}$ in smokers without chronic obstructive pulmonary disease Receiver operating characteristic (ROC) curves for conventional indices – (*A*) ${\dot{V}}_{E}$–${\dot{V}}_{CO_{2}}$ slope and (*B*) ${\dot{V}}_{E}$/${\dot{V}}_{CO_{2}}$ nadir – compared with the novel $\eta {\dot{V}}_{E}$ (*C*) in discriminating smokers with reduced diffusing capacity (DLco < LLN). $\eta {\dot{V}}_{E}$ exhibited the highest diagnostic accuracy (AUC = 0.82; sensitivity 87%, specificity 73%), outperforming both conventional indices (AUC = 0.73 for slope and nadir). Adapted from Muller, P.T. et al. (2021), with permission.

In a cohort of current smokers matched for age, sex and spirometric indices (*n* = 30), individuals with reduced diffusing capacity (DLco < LLN, *n* = 15) exhibited significantly lower $\eta {\dot{V}}_{E}$ values than those with preserved DLco (*P* = 0.004). In contrast, the conventional ${\dot{V}}_{E}$–${\dot{V}}_{CO_{2}}$ slope and ventilatory equivalent nadir did not differ significantly between groups (*P* > 0.05 for both). Receiver operating characteristic analysis demonstrated superior discriminatory performance of $\eta {\dot{V}}_{E}$ (AUC = 0.82), exceeding that of conventional indices (Fig. 10). Thus, $\eta {\dot{V}}_{E}$ was the only variable to retain independent discriminatory value for reduced DLco, supporting the interpretation that the index captures functional gas‐exchange inefficiency rather than age, body size or spirometric scaling alone.

### Smokers with isolated diffusion impairment (*post hoc*)

In a *post hoc* analysis of the same cohort (*n* = 30), $\eta {\dot{V}}_{E}$ remained the only significant predictor of diffusion impairment (*P* = 0.016), whereas MVV_pred showed no significant association. Other covariates, including age, sex and height, were not associated with DLco status (Table 6).

**Table 6:** *Post hoc* analysis. Multivariate predictors of reduced diffusing capacity

Variable/ model	AUC	*P‐*value	*P‐*values multivariate model
$\eta {\dot{V}}_{E}$ (%)	0.822	**0.015**	**0.016**
Age (years)	0.527	0.843	0.296
Height (cm)	0.542	0.800	0.205
Sex (M/F)	0.567	0.465	0.336
MVV_pred (FEV1_pred _*_ 41, L)	0.547	0.707	0.586
Multiple model	0.840	–	–

*Notes*: Outcome coded as 1 = reduced diffusing capacity and 0 = normal. MVV_calc was estimated as FEV_1__predicted × 41. Coefficients represent log‐odds estimated by maximum likelihood. Statistical significance was defined as *P* < 0.05. Model discrimination was assessed by the area under the ROC curve (AUC). Pseudo‐*R* ^2^ (McFadden) = 0.27. $\eta {\dot{V}}_{E}$ remained the only independent predictor after multivariable adjustment.

Abbreviations: AUC, area under the curve; $\eta {\dot{V}}_{E}$_,_ ventilatory efficiency index; FEV_1_, forced expiratory volume in one second; MVV, maximal voluntary ventilation.

### Hyperventilation under heightened ventilatory drive

**Figure 11:**
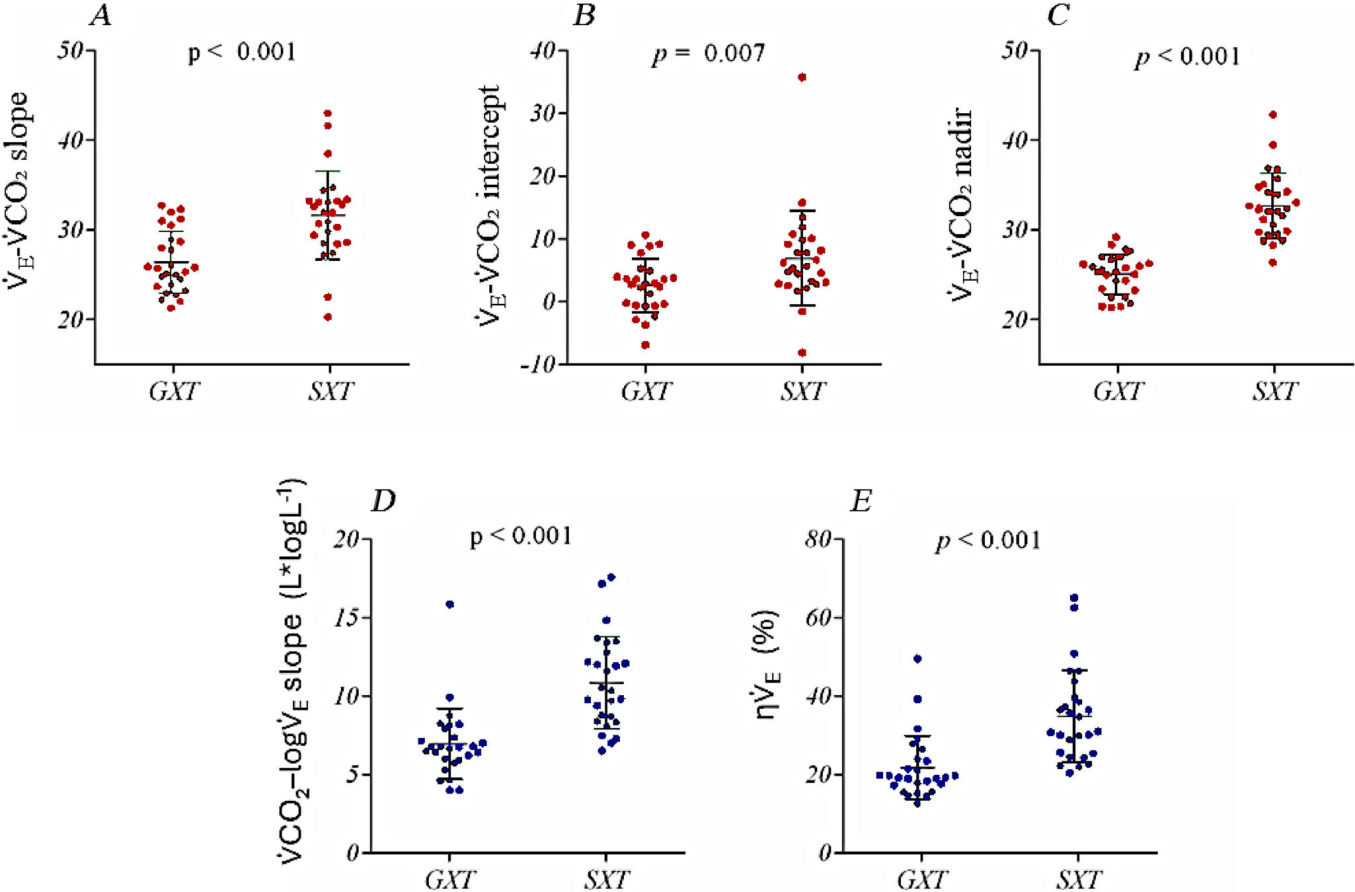
Comparison of ventilatory efficiency metrics in athletes performing graded exercise tests (GXT) and supramaximal constant‐work rate (SXT) bouts Traditional indices – including the ${\dot{V}}_{E}$–${\dot{V}}_{CO_{2}}$ slope, intercept and nadir – increased significantly during SXT, potentially suggesting ventilatory inefficiency. In contrast, the novel semi‐logarithmic indices (${\dot{V}}_{CO_{2}}$–log ${\dot{V}}_{E}$ slope and $\eta {\dot{V}}_{E}$) indicated higher efficiency, consistent with enhanced CO_2_ clearance under elevated ventilatory drive. Adapted from Barbosa, J.P.A. et al. (2024), with permission.

In athletes, supramaximal exercise test (SXT) induced a disproportionate increase in ventilation relative to CO_2_ output, resulting in steeper ${\dot{V}}_{E}$–${\dot{V}}_{CO_{2}}$ slopes compared with graded exercise (GXT) (Fig. 11*A*). Of note, approximately half of the participants exhibited ${\dot{V}}_{E}$–${\dot{V}}_{CO_{2}}$ nadir values ≥34 during SXT, despite preserved gas‐exchange and cardiorespiratory performance. In contrast, both the ${\dot{V}}_{CO_{2}}$–log ${\dot{V}}_{E}$ slope and $\eta {\dot{V}}_{E}$ increased consistently under SXT conditions, indicating improved ventilatory efficiency despite elevated ventilatory drive (Fig. 11*D* and *E*).

This distinction is physiologically important: $\eta {\dot{V}}_{E}$ does not simply track the magnitude of ventilation, but rather the efficiency with which ventilatory recruitment is converted into CO_2_ clearance relative to individualized capacity.

## Discussion

### Main findings

The main finding of this study is that $\eta {\dot{V}}_{E}$ behaves as a normalized index of ventilatory efficiency: it remains relatively stable across healthy adulthood because empirical ventilatory performance and theoretical ceiling decline proportionally, yet it becomes meaningfully altered when gas‐exchange efficiency is functionally impaired or ventilatory drive is disproportionately increased. In the present population‐based cohort, $\eta {\dot{V}}_{E}$ showed minimal dependence on age, sex and spirometric scaling, indicating that it is not reducible to conventional morphometric or ventilatory determinants. Deterministic and regression‐based analyses suggest that this apparent invariance arises from proportional coupling between observed ventilatory behaviour and structural ventilatory capacity. Accordingly, $\eta {\dot{V}}_{E}$ may be interpreted as an emergent physiological property reflecting coordinated interactions among ventilatory control, gas exchange and mechanical constraints, consistent with integrative perspectives in respiratory physiology (Macklem, 2008). Operationally, this implies that $\eta {\dot{V}}_{E}$ captures the integrated outcome of how ventilatory demand is translated into CO_2_ clearance relative to the individualized theoretical ceiling, preserving interpretive coherence across distinct physiological conditions. This behaviour was confirmed across distinct physiological conditions, including diffusion limitation and heightened ventilatory drive.

### Interpreting efficiency during the lifespan

The ${\dot{V}}_{CO_{2}}$–log ${\dot{V}}_{E}$ slope decreased with age and was consistently lower in women, indicating that higher ventilation is required for a given CO_2_ output (see Fig. 1*B* for interpretation), consistent with the well‐established age‐ and sex‐related increases in the ${\dot{V}}_{E}$–${\dot{V}}_{CO_{2}}$ slope reported in the literature (Neder et al., 2001; Sun et al., 2002). However, this age‐related decline in ventilatory performance is accompanied by a parallel reduction in predicted ventilatory capacity, resulting in preservation of the relative fraction of ventilatory reserve utilized during exercise. This proportional adjustment explains the near stability of $\eta {\dot{V}}_{E}$ across the lifespan, as the index reflects the relationship between ventilatory demand and available predicted capacity rather than absolute output.

A similar pattern can be considered for peripheral oxygen extraction. Although ageing is associated with reduced convective oxygen delivery, peripheral extraction remains an active component of the integrated oxygen transport response (Betik & Hepple, 2008). Because oxygen extraction behaves as a regulated fractional process approaching a physiological ceiling (∼90–95%) (Skattebo et al., 2020), it is conceptually analogous to $\eta {\dot{V}}_{E}$: both express the fraction of an available physiological reserve recruited to sustain metabolic demand. Thus, modest age‐related increases in either variable do not indicate greater efficiency *per se*, but greater reliance on the remaining ventilatory or circulatory reserve as maximal capacity declines. This pattern reflects a form of ‘adaptive efficiency’, whereby proportional function is maintained through compensatory adjustments that preserve systemic balance as theoretical ceilings decline (Gifford et al., 2016; Koch & Britton, 2008; Weibel et al., 1991). Hence, these indices reflect resilience rather than enhancement, expressing proportionality preserved within physiological limits (Koch & Britton, 2008; Weibel et al., 1991). Within this conceptual framework, the present model appears to approximate a form of efficiency that is maintained not by maximizing output, but by dynamically matching ventilatory demand to structurally constrained capacity under regulatory and informational control (Gnaiger et al., 1998; Macklem, 2008).

Although ageing is accompanied by a decline in peak ${\dot{V}}_{E}$ due to mechanical and structural limitations (Johnson & Dempsey, 1991; Roman et al., 2016), the proportional coupling between ventilatory drive and gas exchange remains largely preserved. Arterial CO_2_ tension is maintained during moderate‐to‐intense exercise with ageing (Brischetto et al., 1984; Williams & Babb, 1997), indicating that effective clearance persists despite reduced ventilatory output. Within this framework, $\eta {\dot{V}}_{E}$ reflects not an increase in efficiency *per se*, but the preservation of proportional coupling between ventilatory demand and gas exchange. Efficiency thus emerges as a regulated, system‐level property, allowing $\eta {\dot{V}}_{E}$ to remain stable across the lifespan despite progressive declines in its underlying structural components.

### Rethinking linearity in ventilatory coupling

The semi‐logarithmic ${\dot{V}}_{CO_{2}}$–log ${\dot{V}}_{E}$ approach provides a complementary perspective to conventional linear analyses by focusing on the post‐VT_1_ domain, where ventilatory responses become increasingly nonlinear (Phillips et al., 2020; Whipp et al., 1982). Traditional ${\dot{V}}_{E}$–${\dot{V}}_{CO_{2}}$ slopes, while clinically useful, impose linear assumptions on an inherently exponential process shaped by metabolic acidosis, chemoreflex activation and mechanical constraints (Johnson & Dempsey, 1991; Ramos et al., 2013). By transforming ventilation logarithmically, the present framework restores linearity to this domain (Muller, 2023), enabling consistent slope estimation and revealing differences in ventilatory behaviour that are not apparent under conventional analysis. As illustrated in Fig. 7, individuals with identical ${\dot{V}}_{E}$–${\dot{V}}_{CO_{2}}$ slopes may exhibit substantially different ventilatory–metabolic coupling when analysed using the semi‐logarithmic approach. This suggests that the ${\dot{V}}_{CO_{2}}$–log ${\dot{V}}_{E}$ slope captures physiologically relevant information that is partially obscured by linear models, particularly under conditions of elevated ventilatory drive.

### Scale independence and proportional ventilatory–metabolic coupling

The sensitivity analysis further clarifies the role of model assumptions in shaping $\eta {\dot{V}}_{E}$. Joint variation of MVV scaling and FECO_2__max produced predictable proportional shifts in absolute $\eta {\dot{V}}_{E}$ values, without altering the correlation structure with FEV_1_ or the relative ordering of distribution percentiles. This indicates that these parameters primarily influence calibration rather than physiological meaning. In practical terms, $\eta {\dot{V}}_{E}$ behaves as a ratio‐based construct whose interpretive value is preserved across plausible variations in its defining constants, reinforcing its robustness as a descriptor of ventilatory–metabolic coupling rather than a function of arbitrary scaling choices.

Deterministic modelling showed that the apparent age neutrality of $\eta {\dot{V}}_{E}$ emerges from the proportional decline of both the empirical ${\dot{V}}_{CO_{2}}$–log ${\dot{V}}_{E}$ slope and its denominator. This supports a mechanistic validation approach, in which model behaviour is tested against physiological principles rather than statistical associations alone (Viceconti & Hunter, 2016; Viceconti et al., 2020). Accordingly, the stability of $\eta {\dot{V}}_{E}$ reflects intrinsic ventilatory–metabolic coupling, reinforcing its interpretation as a physiologically derived variable with generalizable and causally coherent properties (Hester et al., 2011).

Although age, sex and body size influence the absolute behaviour of ventilatory variables, their effects are largely embedded within the structural components of the $\eta {\dot{V}}_{E}$ formulation – particularly through MVV_pred. As a result, these variables contribute to the calibration of normative equations but exert minimal residual influence once the index is expressed as a ceiling‐normalized ratio. This distinction explains why demographic variables are retained in the derivation of reference percentiles yet show limited explanatory power in regression models. The low proportion of explained variance (∼8.5%) further indicates that most variability in $\eta {\dot{V}}_{E}$ arises from integrative physiological processes rather than from anthropometric structure. From a modelling perspective, this behaviour is consistent with allometric normalization, in which dimensionless ratios reduce geometric sources of variability and emphasize functional relationships (Nevill & Holder, 1995; Weisberg, 2014).

### Construct validation across physiological extremes

The discriminative performance of $\eta {\dot{V}}_{E}$ in diffusion‐limited conditions supports its physiological specificity. In smokers without resting airflow obstruction, $\eta {\dot{V}}_{E}$ was reduced in individuals with impaired DLco despite similar ceiling‐based denominators compared with controls, indicating that differences arise from altered ventilatory–metabolic coupling rather than algebraic scaling. This pattern may reflect early, subclinical exercise airflow limitations, enhancing the sensitivity of $\eta {\dot{V}}_{E}$ to subtle disruptions in ventilatory control before overt obstruction becomes detectable (Muller et al., 2021).

Conversely, in athletes, conventional indices may suggest ventilatory inefficiency due to elevated ventilatory drive, whereas $\eta {\dot{V}}_{E}$ remains physiologically coherent by accounting for nonlinear ventilatory expansion. This resolves a known paradox in exercise physiology, where highly trained individuals may exhibit steep ${\dot{V}}_{E}$–${\dot{V}}_{CO_{2}}$ slopes without true inefficiency (Petek et al., 2022). This paradox has fuelled diagnostic uncertainty, including the risk of overinterpreting steeper slopes as latent cardiomyopathy or maladaptive ventilatory responses (Barbosa et al., 2024; Guazzi, 2025; Husaini & Emery, 2024; Mazaheri et al., 2021; McHugh et al., 2025). The limitation arises from the linear structure of the ${\dot{V}}_{E}$–${\dot{V}}_{CO_{2}}$ relationship itself: as ventilatory drive and tidal flow expand disproportionately, the slope may steepen algebraically without any true loss of efficiency.

Across these contrasting conditions, $\eta {\dot{V}}_{E}$ consistently reflects the balance between ventilatory effort and effective CO_2_ clearance, reinforcing its validity as an integrative index.

### Boundaries and methodological considerations

The interpretation of $\eta {\dot{V}}_{E}$ should be considered within the assumptions used to define the theoretical ventilatory ceiling. In the present study, ventilatory capacity was approximated using a constant‐based formulation (MVV_pred = FEV_1_ × 41) and a fixed value for maximal expired CO_2_ fraction (FECO_2__max = 0.22). While these assumptions provide practical simplicity and reproducibility, they represent simplified approximations of complex physiological limits. Sensitivity analyses demonstrated that variation of these parameters within physiologically plausible ranges produced proportional changes in absolute $\eta {\dot{V}}_{E}$ values, without altering the correlation structure with FEV_1_ or the relative ordering of distribution percentiles. This indicates that the index is primarily influenced by scaling in absolute terms, whereas its physiological interpretation remains stable across alternative assumptions. From a methodological perspective, the definition of predicted ventilatory capacity represents an additional consideration. Although constant‐based approximations facilitate implementation, future normative studies may benefit from incorporating population‐specific predictive equations for MVV_pred to refine calibration, without affecting the underlying physiological meaning of $\eta {\dot{V}}_{E}$. Importantly, these findings support the interpretation that the discriminative and interpretive value of $\eta {\dot{V}}_{E}$ is preserved across plausible variations in model assumptions.

### Translational perspectives

CPET is widely used to integrate cardiovascular, ventilatory and metabolic responses during exercise, supporting diagnosis, prognosis and therapeutic decision‐making (Arena et al., 2011; Guazzi et al., 2017; Stickland et al., 2022). In routine practice, ventilatory efficiency is primarily interpreted through the ${\dot{V}}_{E}$–${\dot{V}}_{CO_{2}}$ slope and its nadir. Although robust, these indices may become difficult to interpret when ventilation is either mechanically constrained (Neder et al., 2015; Zuffo et al., 2024) or disproportionately increased relative to metabolic demand (Barbosa et al., 2024; Kasiak et al., 2024; Petek et al., 2022).

In practical terms, this creates a recurrent clinical ambiguity: similar ${\dot{V}}_{E}$–${\dot{V}}_{CO_{2}}$ slopes may reflect fundamentally different physiological states. A relatively ‘normal’ slope can occur in patients with limited ventilatory expansion – such as those with obesity, chronic obstructive pulmonary disease or neuromuscular weakness – not because ventilatory–metabolic coupling is preserved, but because ventilation cannot increase appropriately (Müller et al., 2023). Conversely, elevated slopes in highly trained individuals or in states of heightened ventilatory drive may reflect adaptive hyperventilation rather than inefficiency (McHugh et al., 2025). These situations are familiar to clinicians but are not easily resolved using conventional metrics alone.

The $\eta {\dot{V}}_{E}$ framework addresses this ambiguity by expressing ventilatory performance relative to an individualized theoretical ceiling. From a practical standpoint, this does not require additional testing or new measurements: $\eta {\dot{V}}_{E}$ can be derived directly from standard CPET data using the post‐VT_1_ segment of the ${\dot{V}}_{CO_{2}}$–log ${\dot{V}}_{E}$ relationship and predicted ventilatory capacity. What changes, therefore, is not the test itself, but its interpretation. Clinically, this means that $\eta {\dot{V}}_{E}$ may help distinguish exaggerated ventilatory responses driven by demand from genuinely inefficient ventilatory–gas‐exchange coupling, thereby complementing conventional ${\dot{V}}_{E}$–${\dot{V}}_{CO_{2}}$ metrics in physiological phenotyping and early detection of gas‐exchange limitation.

Importantly, $\eta {\dot{V}}_{E}$ is not intended to localize the primary site of limitation within the integrative oxygen transport pathway (i.e. lung, circulation or skeletal muscle), which remains the role of comprehensive CPET interpretation. Rather, its contribution is to refine the interpretation of the ventilatory component within this integrated response. In this context, $\eta {\dot{V}}_{E}$ does not directly distinguish between specific pulmonary mechanisms such as diffusion limitation *versus* ventilation–perfusion mismatch. However, by expressing ventilatory performance relative to a theoretical ceiling, it helps identify when the ventilatory response is disproportionately low or high relative to metabolic demand. This, in turn, provides indirect physiological insight into whether impaired CO_2_ clearance reflects altered gas‐exchange efficiency, excessive ventilatory drive or mechanical constraint, thereby supporting more informed interpretation rather than acting as a standalone diagnostic classifier.

In daily laboratory practice, this allows a more physiologically consistent reading of CPET in three common scenarios. First, when ventilatory expansion is constrained, $\eta {\dot{V}}_{E}$ may reveal reduced efficiency despite a seemingly preserved ${\dot{V}}_{E}$–${\dot{V}}_{CO_{2}}$ slope, helping to avoid underestimation of disease severity. Second, in conditions of excessive ventilatory drive, $\eta {\dot{V}}_{E}$ can distinguish adaptive increases in ventilation from true impairment in CO_2_ clearance, reducing the risk of overinterpretation. Third, in patients with unexplained exertional dyspnoea, $\eta {\dot{V}}_{E}$ provides an additional layer of interpretation by clarifying whether the observed ventilatory pattern reflects altered gas exchange, ventilatory control or mechanical limitation.

Accordingly, the clinical contribution of $\eta {\dot{V}}_{E}$ is not to replace established CPET indices, but to refine their interpretation in situations where proportional metrics alone become ambiguous. Because it is derived from routinely acquired variables, it can be readily implemented in clinical laboratories without changes in protocol. Its potential value lies in improving physiological phenotyping and reducing interpretive uncertainty across heterogeneous conditions, particularly when conventional ventilatory efficiency measures appear discordant with the overall clinical picture.

At present, $\eta {\dot{V}}_{E}$ should be regarded as a physiology‐informed adjunct within CPET interpretation. Broader clinical adoption will depend on further validation in populations where ventilatory constraint, diffusion impairment and circulatory limitation coexist. Within the context of a Techniques paper, its principal contribution is to provide a methodologically grounded framework that enables both more precise physiological interpretation and the experimental testing of hypotheses related to ventilatory–metabolic coupling across health and disease.

## Conclusions

This study demonstrates that ventilatory efficiency can be more accurately quantified using a semi‐logarithmic framework focused on the post‐threshold ventilatory response. By normalizing CO_2_ clearance to a theoretical ceiling based on predicted ventilatory capacity, $\eta {\dot{V}}_{E}$ provides a scale‐independent measure of ventilatory efficiency, showing minimal sensitivity to age, sex or spirometric factors. The ceiling‐based formulation offers a complementary perspective to existing physiological models, in which performance can be interpreted relative to boundary conditions, providing an additional reference context. By preserving interpretability within a bounded scale, $\eta {\dot{V}}_{E}$ maintains conceptual clarity while remaining adaptable to diverse physiological applications.

## Additional information

## Competing interests

The authors declare that the research was conducted in the absence of any commercial or financial relationships that could be construed as a potential conflict of interest.

## Author contributions

Conception and design: P.T.M. and R.E., with P.T.M., R.E. and J.A.N. writing the first draft. Analysis and/or interpretation: all authors, with T.I. and E.S.F. taking primary responsibility for the statistical analyses. Drafting the manuscript for important intellectual content: all authors.

## Funding

Paulo de Tarso Müller was supported by a productivity research grant PQ‐2 from the Brazilian National Council for Scientific and Technological Development. Grant number 302812/2022‐9 (CNPq). Ralf Ewert was supported by a grant from the German Federal Ministry of Education and Research. Grant number 01ZZ9603.

## Supporting information


Peer Review History

## Data Availability

The complete regression‐based validation dataset, anonymized raw data tables, and all analysis scripts used in this study are openly available in Zenodo (https://doi.org/10.5281/zenodo.17508654). Additional supporting materials are provided in Appendices A and B. Further data may be obtained from the corresponding author upon reasonable request.

## Appendix A

### Physiological rationale for the $F_{\mathrm{EC}O_{2}}$ ceiling (≈ 0.22)

The maximal theoretical fraction of alveolar CO_2_ in dry gas can be derived from the alveolar gas equation (West & Luks, 2016; West et al., 2007), expressed as P_AO_2_ = (P_atm − P_H_2_O) × F_IO_2_ − P_ACO_2_ / R, where P_AO_2_ is the alveolar O_2_ partial pressure, P_atm the atmospheric pressure (≈ 760 mmHg at sea level), P_H_2_O the water vapour pressure (≈ 47 mmHg at 37°C), F_IO_2_ the inspired O_2_ fraction (∼0.21), P_ACO_2_ the alveolar CO_2_ partial pressure, and *R* the respiratory quotient (≈1.0 at rest, increasing to ∼1.1–1.2 during intense exercise).

At the assumed theoretical limit of complete O_2_ extraction (P_AO_2_ → 0), the equation simplifies to P_ACO_2_, max = *R* × (P_atm − P_H_2_O) × F_IO_2_. Substituting standard sea‐level values yields P_ACO_2_, max ≈ *R* × (760 − 47) × 0.21 ≈ *R* × 149.7 mmHg. Accordingly, this corresponds to a maximal CO_2_ fraction (F_ACO_2_, max) of approximately 0.21 for *R* = 1.0, ∼0.23 for *R* = 1.1, and ∼0.25 for *R* = 1.2.

Accordingly, adopting FECO_2_, max ≈ 0.22 provides a conservative and physiologically grounded upper bound. This assumes near‐complete O_2_ uptake, with nitrogen (∼78% of dry gas) acting as an inert background.

Importantly, typical physiological values of expired or alveolar CO_2_ (e.g. $ET_{CO_{2}}$ or P_AECO_2_) are much lower (∼0.04–0.06). Thus, 0.22 should be interpreted strictly as a theoretical ceiling, not a measurable norm. In this context, the value represents a boundary condition derived from a limiting physiological scenario rather than an empirical average, and the use of 0.22 as the denominator constant in $\eta {\dot{V}}_{E}$ anchors the index to a biologically plausible upper bound, enhancing interpretability without affecting its structural meaning.

## Appendix B

### Algorithm box – computation of $\eta {\dot{V}}_{E}$ from CPET

This procedure describes how to compute $\eta {\dot{V}}_{E}$ as a normalized index of ventilatory efficiency from breath‐by‐breath or time‐averaged CPET data, using the semi‐logarithmic ${\dot{V}}_{CO_{2}}$–log ${\dot{V}}_{E}$ domain. The required inputs include ${\dot{V}}_{E}$ and ${\dot{V}}_{CO_{2}}$ (averaged over 5–10 s, expressed in body temperature and pressure, saturated (BTPS) and standard temperature pressure, dry (STPD) units), the first ventilatory threshold (VT_1_), and a measure of predicted ventilatory capacity derived either from FEV_1__pred or directly from MVV_pred (commonly approximated as constant × FEV_1__pred). The computation also relies on fixed constants, with FECO_2__max ≈ 0.22 and R_mix ≈ 0.826, yielding *k_ref* ≈ 0.182.

The output consists of the empirical slope (*b_emp*) of the ${\dot{V}}_{CO_{2}}$ *versus* log_10_ ${\dot{V}}_{E}$ relationship, the theoretical reference slope (*b_ref*), defined as MVV_pred × *k_ref*, and the resulting $\eta {\dot{V}}_{E}$ expressed as $\eta {\dot{V}}_{E}$ (%) = 100 × (*b_emp / b_ref*). Standard quality‐control metrics should also be reported, including *R* ^2^, number of data points, residual distribution and identification of outliers.

In practice, the analysis begins with alignment and cleaning of the data, excluding non‐physiological values. Signals are then smoothed, and the data are binned into 5–10 s epochs using the median. The analysis window is restricted from VT_1_ to peak exercise, ensuring at least 30 data points. The independent variable is transformed as *x* = log_10_ ${\dot{V}}_{E}$, and the relationship ${\dot{V}}_{CO_{2}}$ = a + *b_emp*·x is fitted using ordinary least squares. Model quality is assessed through *R* ^2^, and outliers (residuals > 3 standard deviations) are removed once before refitting. The reference slope is then computed as *b_reF* = MVV_pred × FECO_2__max × R_mix, and $\eta {\dot{V}}_{E}$ is calculated together with its 95% confidence interval. Final quality‐control criteria include *R* ^2^ ≥ 0.60, *N* ≥ 30, and stability of the slope (variation <10%).

For minimal reporting, it is recommended to provide the number of points (*N*), bin size, *R* ^2^, the estimated slope (b_emp ± SE), $\eta {\dot{V}}_{E}$ (% ± 95% CI), the constants adopted (FECO_2__max, R_mix, k_ref), and any quality‐control flags.
