# Supplementary material for: Ventilatory efficiency: Physiological modelling and mechanistic validation
Source: J Physiol. 2026 Jun 17;604(14):5777–99. doi: 10.1113/JP290456 (PMC13370739; doi:10.1113/JP290456)
Supplement: Supplementary file 1 — Peer Review History [file TJP-604-5777-s001.pdf]

The Journal of Physiology

Ventilatory Efficiency: Physiological Modelling and Mechanistic Validation

PAULO de TARSO MÜLLER, BEATE STUBBE, TILL ITTERMANN, ERLANDSON FERREIRA SARAIVA, J. Alberto Neder, and RALF EWERT
DOI: 10.1113/JP290456

Corresponding author(s): PAULO MÜLLER (paulo.muller@ufms.br)

The referees have opted to remain anonymous.

Review Timeline:

Submission Date:	03-Nov-2025
Editorial Decision:	16-Dec-2025
Revision Received:	17-Apr-2026
Editorial Decision:	06-May-2026
Revision Received:	11-May-2026
Accepted:	20-May-2026

Senior Editor: Paul Greenhaff

Reviewing Editor: Frank Powell

Transaction Report:

(Note: With the exception of the correction of typographical or spelling errors that could be a source of ambiguity, letters and reports are not edited. Depending on transfer agreements, referee reports obtained elsewhere may or may not be included in this compilation. Referee reports are anonymous unless the Referee chooses to sign their reports.)

Dear Dr MÜLLER,

Re: JP-TFP-2025-290456 "**Ventilatory Efficiency: Physiological Modelling and Mechanistic Validation**" by PAULO de TARSO MÜLLER, BEATE STUBBE, TILL ITTERMANN, ERLANDSON FERREIRA SARAIVA, J. Alberto Neder, and RALF EWERT

Thank you for submitting your manuscript to The Journal of Physiology. It has been assessed by a Reviewing Editor and by 2 expert referees and we are pleased to tell you that it is potentially acceptable for publication following satisfactory major revision.

Please advise your co-authors of this decision as soon as possible.

The referee reports are copied at the end of this email.

Please address all the points raised and incorporate all requested revisions or explain in your Response to Referees why a change has not been made. We hope you will find the comments helpful and that you will be able to return your revised manuscript within 9 months. If you require longer than this, please contact journal staff: jp@physoc.org. Please note that this letter does not constitute a guarantee for acceptance of your revised manuscript.

Your revised manuscript should be submitted online using the link in your Author Tasks: Link Not Available. This link is accessible via your account as Corresponding Author; it is not available to your co-authors. If this presents a problem, please contact journal staff (jp@physoc.org). Image files from the previous version are retained on the system. Please ensure you replace or remove any files that are being revised.

If you do not wish to submit a revised version of your manuscript, you must inform our journal staff (jp@physoc.org) or reply to this email to request withdrawal. Please note that a manuscript must be formally withdrawn from the peer review process at one journal before it may be submitted to another journal.

TRANSPARENT PEER REVIEW POLICY: To improve the transparency of its peer review process, The Journal of Physiology publishes online as supporting information the peer review history of all articles accepted for publication. Readers will have access to decision letters, including Editors' comments and referee reports, for each version of the manuscript, as well as any author responses to peer review comments. Referees can decide whether or not they wish to be named on the peer review history document.

ABSTRACT FIGURES: Authors are expected to use The Journal's premium BioRender account to create/redraw their Abstract Figures. Information on how to access this account is here: https://physoc.onlinelibrary.wiley.com/journal/14697793/biorender-access.

This will enable Authors to create and download high-resolution figures. If authors have used the free BioRender service, they can use the instructions provided in the link above to download a high-resolution version suitable for publication.

The link provided should only be used for the purposes of this submission. Authors will be charged for figures created on this account if they are not related to this manuscript submission.

LANGUAGE EDITING AND SUPPORT FOR PUBLICATION: If you would like help with English language editing, or other article preparation support, Wiley Editing Services offers expert help, including English Language Editing, as well as translation, manuscript formatting, and figure formatting at www.wileyauthors.com/eeo/preparation. You can also find resources for Preparing Your Article for general guidance about writing and preparing your manuscript at www.wileyauthors.com/eeo/prepresources.

RIGOUR AND REPRODUCIBILITY: We recommend authors consult The Journal's Rigour and Reproducibility requirements to ensure that their manuscript is suitable for publication: https://physoc.onlinelibrary.wiley.com/pb-assets/hub-assets/physoc/documents/TJP-Rigour-and-Reproducibility-Requirements.pdf

REVISION CHECKLIST:

Check that your Methods section conforms to journal policy: https://jp.msubmit.net/cgi-bin/main.plex?form_type=display_requirements#methods.

Check that data presented conforms to the statistics policy: https://jp.msubmit.net/cgi-bin/main.plex?form_type=display_requirements#statistics.

Upload a full Response to Referees file. To create your 'Response to Referees': copy all the reports, including any comments from the Senior and Reviewing Editors, into a Microsoft Word, or similar, file and respond to each point, using font or background colour to distinguish comments and responses and upload as the required file type.

Please upload two versions of your manuscript text: one with all relevant changes highlighted and one clean version with no changes tracked. The manuscript file should include all tables and figure legends, but each figure/graph should be uploaded as separate, high-resolution files. The journal is now integrated with Wiley's Image Checking service. For further details, see: https://www.wiley.com/en-us/network/publishing/research-publishing/trending-stories/upholding-image-integrity-wileys-image-screening-service

You may also upload:

- 'Potential Cover Art' for consideration as the issue's cover image
- Appropriate Supporting Information (Video, audio or data set: see https://jp.msubmit.net/cgi-bin/main.plex?form_type=display_requirements#supp).

We look forward to receiving your revised submission.

If you have any queries, please reply to this email and we will be pleased to advise.

Yours sincerely,

Paul Greenhaff
Senior Editor
The Journal of Physiology

REQUIRED ITEMS

- Please upload separate high-quality figure files via the submission form.

- Please ensure that any tables are editable and in Word format, and wherever possible, embedded in the article file itself.

- Please ensure that the Article File you upload is a Word file.

EDITOR COMMENTS

Reviewing Editor:

Comments to the Author:
Your work has been reviewed by two experts and they both have questions and issues that need to be addressed. The size and detail of the study is appreciated but reviewers comment on the need for streamlining the message and simplifying the paper. Discussions of asymptotic relations in nature and log transformations in biomedical research are two (small) opportunities I notice in just the Introduction.

Something I noticed (that relates to some of Rev. 1's comments) is that the Translational Perspectives could be more explicit on exactly how exercise testing is used clinically. Is the goal of this measure to identify a limitation in the lung versus heart, circulation or muscle? Presumably it would identify the lung but is it also useful for diagnosing the mechanisms, e.g. diffusion versus V/Q mismatching, or designing a treatment? Considering that this is a Techniques paper, the point is to emphasize the method and justify its use and development, to either experimentally test hypotheses for physiological mechanisms (in this particular journal) or for clinical use.

Senior Editor:

Comments to the Author:
Thank you for the Techniques for Physiology manuscript submission to The Journal of Physiology. The manuscript has been considered by a reviewing editor and two expert reviewers. All were positive about the significant number of volunteers studied. However, in its present form the manuscript is not acceptable for publication. In short, it is viewed as being somewhat impenetrable for the readership of The Journal of Physiology and needs to be significantly shortened, simplified and the central message made clearer. Please also note The Journal of Physiology does not usually accept

supplemental/supporting material that cannot be included with the body of the manuscript. Please read The Journal's policy regarding Supporting Information. https://jp.msubmit.net/cgi-bin/main.plex?form_type=display_requirements#suppinfo

Please consider the points raised by the Reviewing Editor and expert reviewers in detail. We look forward to receiving a revised version of the manuscript and rebuttal document.

REFEREE COMMENTS

Referee #1:

The authors propose a new approach to evaluate ventilatory efficiency in exercising humans. The manuscript is very articulated, complex and detailed, and I admit that I did not understand many aspects of data analysis and interpretation. I am afraid that this would apply also to many others "average readers" of the Journal.

In any case, the manuscript is well written and organized. The research group is expert in the field. The number of subjects (1150 !) is impressive.

According to the authors, the standard methods actually utilized to evaluate ventilatory efficiency (V'E/V'CO2 slope, V'E/V'CO2 nadir, etc.) deal mainly (or only) with sub-ventilatory threshold exercise, and "miss" the more physiologically relevant exponential increase of the variable occurring above the first ventilatory threshold and above the "respiratory compensation point". I totally agree with this concept.

Thus, they propose a semi-logarithmic approach to data analysis (V'CO2 vs log V'E) which linearizes the exponential segment of the ventilatory response. Again, I would totally support this approach. A linear slope of a variable is much easier to handle and interpret vs an exponential increase. By incidence, are they sure that no one proposed this semi-logarithmic approach before?

The authors, however, loose me when they propose the next step: they "normalize" the slope of the linear V'CO2/log V'E increase to a "theoretical physiological limit for gas exchange", "scaled to the predicted maximal voluntary ventilation (MVV) and the theoretical limit of expired CO2 fraction". This normalization yields a "bounded efficiency index" (ηV'E), expressed as a percentage. The resulting ηV'E was "robustly stable and only weakly associated with age and lung function". But, what is the physiological significance of a ventilatory efficiency parameter which does not change with age or lung function? I am sorry, I probably completely missed the point, but I do not understand the meaning and the relevance of this variable.

If I have a sick lung, then V'CO2/logV'E slope would be lower. But, also the estimated MVV would be lower. Then the bounded efficiency index ηV'E would be unchanged vs "normal" subjects. Would the variable evaluate "ventilatory efficiency"? What am I missing?

The study is, in my opinion, too long and complex. A lot of "supplementary material" is proposed. In my opinion the authors should make a real effort to shorten and simplify the manuscript. Please remember that the study should say something also to an "average reader" of the Journal.

MVV was estimated, and not measured. The same for the "theoretical limit of expired CO2 fraction". Do these estimations represent a problem? The resulting variables seem quite relevant for the purposes of the study.

Line 120. "maximal"?

Referee #2:

Muller and colleagues propose an interesting new ventilatory efficiency index (ηVE) derived from the slope of the VCO_2-log VE relationship, normalized against a theoretical ventilatory ceiling based on predicted MVV. The use of a large, carefully characterized reference cohort and external validation samples is a strength, and the work appears novel. This is an important contribution to the literature. I only have minor comments and edits.

I understand the need to provide as much detail as possible for a methodological manuscript; however, the manuscript is extremely long and sometimes difficult to follow. Can the authors shorten it and put non-essential text/data in the supplement?

Robustness checks were performed using alternative MVV constants (35, 41, 45), which is reassuring, but the results of these checks are not shown. A sensitivity table quantifying how these assumptions affect ηVE percentiles would be helpful.

In addition, $FECO_2$_max is fixed at 0.22 throughout. Since this parameter contributes directly to the theoretical denominator, a brief sensitivity analysis varying $FECO_2$_max within physiologically plausible limits would help confirm the stability of the index.

P5L135: "Both could misclassified" should be "Both could be misclassified"

P7L194: "to reinterprets and extends" should be "to reinterpret and extend"

P9L258: For maximal exercise criteria, the authors said "attainment of predicted maximal heart rate". Please be more specific. For example, was it {greater than or equal to} 100% predicted? Also, how was predicted max HR determined?

P9L264: SHIP was already defined earlier in the manuscript.

P10L281: Why use FEV1/FVC <0.70 instead of < LLM?

P14L385: The figure was reproduced with permission but I don't see the original citation.

P16L429: Should the authors include the actual constant they used here?

P16L433: I'm not sure it is entirely correct to say that achieving predicted MVV is "physiologically unattainable". Sometimes VEmax can exceed predicted MVV in athletes and clinical populations for a variety of reasons (e.g. exercise-induced bronchodilation, among other reasons).

P19L506: Can the authors clarify why they ultimately decided on 41 as the constant for predicting MVV from FEV1?

Table 1 is missing units for VO2. Also, RER should be reported to 2 decimal places.

Figure 7 is very informative.

In general, figure resolution is quite poor. You can really notice this when you zoom in on the figures. I imagine it won't look great in final published form. The authors are encouraged to improve figure resolution.

END OF COMMENTS

Dear Professor Greenhaff,
Dear Reviewing Editor,

We sincerely thank you and the referees for the careful evaluation of our manuscript (JP-TFP-2025-290456) and for the constructive comments provided. We have undertaken a comprehensive revision and submit here a substantially revised version of the manuscript.

It was reduced from approximately 9500 to 6120 words, with a reorganization of the overall structure to improve logical flow and readability. The Introduction was condensed and refocused on the central physiological problem, and conceptual sections were reformulated to enhance clarity while preserving their full theoretical content. The central methodological figure (Figure 5), which represents the core of the proposed framework, was completely redesigned to allow direct and intuitive understanding of the method, with simplification of supporting equations and assumptions where appropriate. The Graphical Abstract and all figures were reformatted and exported in high resolution according to Journal standards, and figure legends were rewritten to be more self-explanatory. The definition, presentation, and physiological interpretation of the $\eta\dot{V}_E$ index were clarified throughout the manuscript, and the Translational Perspectives section was revised to improve clinical applicability. Sensitivity analyses were incorporated into the main text, supporting material was minimized and integrated, and terminology was standardized across the manuscript.

Point-by-Point Response to Referees regarding:

JP-TFP-2025-290456 "Ventilatory Efficiency: Physiological Modelling and Mechanistic Validation" by PAULO de TARSO MÜLLER, BEATE STUBBE, TILL ITTERMANN, ERLANDSON FERREIRA SARAIVA, J. Alberto Neder, and RALF EWERT.

REFEREE COMMENTS / Referee #1

The authors propose a new approach to evaluate ventilatory efficiency in exercising humans. The manuscript is very articulated, complex and detailed, and I admit that I did not understand many aspects of data analysis and interpretation. I am afraid that this would apply also to many others "average readers" of the Journal.

We thank the reviewer for this important and candid observation. We fully agree that the clarity of presentation in the original version was insufficient, particularly for readers not already familiar with the conceptual framework. In the revised manuscript, we have substantially restructured and condensed the text to improve accessibility and logical flow. Key conceptual steps have been reformulated with the specific aim of making the physiological rationale more transparent to the general readership, while preserving the analytical content. In addition, the central methodological framework has been visually clarified through a complete redesign of Figure 5, which now provides a direct and self-contained representation of the method.

In any case, the manuscript is well written and organized. The research group is expert in the field. The number of subjects (1150 !) is impressive.

We appreciate these encouraging remarks and the recognition of both the cohort size and the experience of the research group.

According to the authors, the standard methods actually utilized to evaluate ventilatory efficiency (V'E/V'CO2 slope, V'E/V'CO2 nadir, etc.) deal mainly (or only) with sub-ventilatory threshold exercise, and "miss" the more physiologically relevant exponential increase of the variable occurring above the first ventilatory threshold and above the "respiratory compensation point". I totally agree with this concept

We are pleased that the reviewer agrees with this central premise. This limitation of conventional indices in the post-VT_1 domain was in fact one of the main motivations for the present work. The revised manuscript now states this rationale more directly and links it more clearly to the need for an alternative framework.

Thus, they propose a semi-logarithmic approach to data analysis (V'CO2 vs log V'E) which linearizes the exponential segment of the ventilatory response. Again, I would totally support this approach. A linear slope of a variable is much easier to handle and interpret vs an exponential increase. By incidence, are they sure that no one proposed this semi logarithmic approach before?

We are glad that the reviewer supports this aspect of the approach. The semi-logarithmic transformation was adopted precisely to enable a more stable and interpretable characterization of the ventilatory response in the post-threshold domain, which is central to the present framework. Semi-logarithmic transformations are indeed widely used in physiology to linearize exponential relationships. However, to the best of our knowledge, the specific formulation adopted here—relating $\dot{V}CO_2$ to log $\dot{V}_E$ in the post-VT_1 domain with the explicit aim of characterizing ventilatory efficiency—has not been previously proposed or systematically applied in this context.

The authors, however, loose me when they propose the next step: they "normalize" the slope of the linear V'CO2/log V'E increase to a "theoretical physiological limit for gas exchange", "scaled to the predicted maximal voluntary ventilation (MVV) and the theoretical limit of expired CO2 fraction". This normalization yields a "bounded efficiency index" (ηV'E), expressed as a percentage. The resulting ηV'E was "robustly stable and only weakly associated with age and lung function". But, what is the physiological significance of a ventilatory efficiency parameter which does not change with age or lung function? I am sorry, I probably completely missed the point, but I do not understand the meaning and the relevance of this variable.

This is a central and legitimate question, and it points to the core physiological meaning of $\eta \dot{V}_E$. The apparent stability of the index across age should not be interpreted as lack of sensitivity, but as evidence of preserved coupling between ventilatory demand and ventilatory capacity in healthy individuals. With ageing, both the ventilatory response to CO_2 and the predicted ventilatory capacity (MVV_pred) decline in parallel, reflecting coordinated changes in lung mechanics, gas-exchange surface, and ventilatory control. As a result, the ratio between these

two components remains relatively stable, not because physiology is unchanged, but because the system scales proportionally. This behavior is consistent with other normalized physiological indices. For example, peak VO_2 expressed as % predicted may remain relatively stable across age, despite substantial declines in absolute oxygen uptake, because both observed and predicted values decrease in parallel. In this context, stability reflects preserved proportional function rather than absence of physiological change.

Crucially, this proportionality is disrupted under specific physiological and pathological conditions, and this is where $\eta\dot{V}_E$ provides additional interpretative value. In our data, the index proved more sensitive than conventional $\dot{V}_E$–$\dot{V}CO_2$ metrics in detecting diffusion-related impairment, identifying reductions in ventilatory efficiency despite preserved spirometry. At the other extreme, in endurance-trained individuals, $\eta\dot{V}_E$ correctly interpreted states of heightened ventilatory drive, distinguishing increased ventilation from true inefficiency. In this context, it resolves a known limitation of conventional indices, which may suggest "inefficiency" under conditions of high ventilatory drive despite preserved gas-exchange coupling.

Thus, $\eta\dot{V}_E$ remains stable when ventilatory demand and capacity are physiologically matched, but deviates when this coupling is disrupted—either by impaired gas exchange or by disproportionate ventilatory drive. In this sense, the index does not reflect absolute ventilatory performance, but the appropriateness of the ventilatory response relative to the individual's physiological capacity.

If I have a sick lung, then V'CO2/logV'E slope would be lower. But, also the estimated MVV would be lower. Then the bounded efficiency index ηV'E would be unchanged vs "normal" subjects. Would the variable evaluate "ventilatory efficiency"? What am I missing

We thank the reviewer for this important and insightful question. With your permission, we would like to clarify this point more directly.

The key point is that the normalization underlying $\eta\dot{V}_E$ does not assume proportional reductions between the ventilatory response and predicted ventilatory capacity—it tests whether such proportionality is preserved. In healthy ageing, these components tend to decline in parallel, and the ratio remains relatively stable, reflecting preserved physiological coupling. However, in disease this proportionality is typically lost. Predicted ventilatory capacity (MVV_pred) represents a normative reference derived from healthy populations, whereas the observed ventilatory response during exercise is influenced by additional factors such as increased dead space, impaired gas exchange, altered chemoreflex drive, and mechanical constraints. These processes are physiologically distinct and do not scale proportionally.

As a result, ventilation may become either disproportionately elevated for a given CO_2 output (e.g. in diffusion impairment), or mechanically constrained despite increased drive (e.g. in COPD), leading to deviations in $\eta\dot{V}_E$. This behavior was observed in our data, where $\eta\dot{V}_E$ identified diffusion-related impairment despite preserved spirometry, while conventional $\dot{V}_E$–$\dot{V}CO_2$ indices showed limited discrimination. Consistent patterns have also been observed across distinct pathophysiological settings, including obstructive and cardiac conditions, supporting that $\eta\dot{V}_E$ captures disruption of ventilatory demand–capacity coupling rather than a mathematical effect of normalization.

Importantly, if proportional scaling were preserved, $\eta\dot{V}_E$ would remain invariant not only across ageing but also across pathological conditions. This was not observed in our data. Instead,

disease states produced systematic deviations in η $\dot{V}_E$, confirming that the index captures disruption of physiological coupling rather than a mathematical consequence of normalization. Therefore, η $\dot{V}_E$ does evaluate ventilatory efficiency, but in a specific sense: it quantifies whether the ventilatory response is appropriate relative to the individual's predicted physiological capacity, rather than reflecting absolute ventilatory output.

The study is, in my opinion, too long and complex. A lot of "supplementary material" is proposed. In my opinion the authors should make a real effort to shorten and simplify the manuscript. Please remember that the study should say something also to an "average reader" of the Journal

We agree with the reviewer's concern regarding length and complexity. The manuscript has been substantially reduced (from approximately 9,500 to 6,120 words) and reorganized to improve clarity and accessibility for the general readership. In particular, conceptual sections were condensed, redundancies removed, and key methodological elements more directly articulated. Selected technical components were relocated to Appendices (e.g. $FECO_2$_max derivation and methodological details), in line with journal guidance, allowing the main text to focus on the physiological rationale and core findings.

MVV was estimated, and not measured. The same for the "theoretical limit of expired CO2 fraction". Do these estimations represent a problem? The resulting variables seem quite relevant for the purposes of the study.

This is an important point. Rather than assuming these estimates to be exact, we explicitly tested their impact on the behavior of the index. Sensitivity analyses incorporating a range of plausible MVV scaling factors and $FECO_2$_max values showed predictable shifts in absolute η $\dot{V}_E$ values, while preserving percentile distribution, rank ordering, and associations with lung function. This indicates that the index is sensitive to scaling in absolute terms but structurally robust in its physiological relationships. In addition, across the validation datasets, η $\dot{V}_E$ consistently demonstrated meaningful discrimination of gas-exchange impairment, supporting that these estimations do not compromise its interpretative value. Additional considerations are now discussed in the Boundaries and Limitations section.

Line 120. "maximal"?

This was an error and has been corrected in the revised manuscript.

REFEREE COMMENTS / *Referee #2*

Muller and colleagues propose an interesting new ventilatory efficiency index (ηVE) derived from the slope of the VCO_2-log VE relationship, normalized against a theoretical ventilatory ceiling based on predicted MVV. The use of a large, carefully characterized reference cohort and external validation samples is a strength, and the work appears novel. This is an important contribution to the literature. I only have minor comments and edits.

We appreciate these constructive and encouraging remarks, as well as the recognition of the novelty of the proposed index and the strength of the cohort and validation strategy.

I understand the need to provide as much detail as possible for a methodological manuscript; however, the manuscript is extremely long and sometimes difficult to follow. Can the authors shorten it and put non-essential text/data in the supplement?

We agree with the reviewer's concern regarding length and readability. The manuscript has been substantially reduced (from approximately 9,500 to 6,120 words) and reorganized to improve clarity and flow. In response to the suggestion to relocate non-essential material, selected technical components were moved to Appendices (e.g. $FECO_2$_max derivation and methodological details), in accordance with journal guidelines. This allowed the main text to focus more directly on the physiological rationale and key findings.

Robustness checks were performed using alternative MVV constants (35, 41, 45), which is reassuring, but the results of these checks are not shown. A sensitivity table quantifying how these assumptions affect ηVE percentiles would be helpful.

This is a helpful suggestion. In response, we have included a dedicated sensitivity analysis (Table 4) quantifying the impact of alternative MVV scaling constants and $FECO_2$_max assumptions on η $\dot{V}_E$ percentiles and model behavior.These analyses show that while absolute η $\dot{V}_E$ values shift predictably with scaling assumptions, percentile distribution, rank ordering, and associations with lung function are preserved, supporting the structural robustness of the index.

This was really helpful, and we thank the insight!

In addition, $FECO_2$_max is fixed at 0.22 throughout. Since this parameter contributes directly to the theoretical denominator, a brief sensitivity analysis varying $FECO_2$_max within physiologically plausible limits would help confirm the stability of the index.

This is a valuable suggestion. In response, we incorporated variation in $FECO_2$_max within a physiologically plausible range into the sensitivity analysis (Table 4), including joint variation with MVV scaling. When varied in isolation, changes in $FECO_2$_max resulted in relatively small shifts in η $\dot{V}_E$ values compared to those observed with MVV scaling, indicating that the index is less sensitive to this parameter. Overall, percentile distribution, rank ordering, and associations with lung function were preserved, supporting the stability of the formulation across plausible assumptions. A concise physiological rationale for the selected $FECO_2$_max value is now provided in Appendix A.

P5L135: "Both could misclassified" should be "Both could be misclassified"

This has been corrected in the revised manuscript.

P7L194: "to reinterprets and extends" should be "to reinterpret and extend"

We appreciate the reviewer for noting this. Corrected in the revised manuscript.

P9L258: For maximal exercise criteria, the authors said "attainment of predicted maximal heart rate". Please be more specific. For example, was it {greater than or equal to} 100% predicted? Also, how was predicted max HR determined?

The criterion follows established practice in large population-based cohorts such as SHIP, where maximal effort is typically defined as attainment of ≥90% of age-predicted maximal heart rate. Predicted values were derived from standard age-based equations commonly used in epidemiological settings. This has now been clarified in the revised manuscript.

P9L264: SHIP was already defined earlier in the manuscript.

Noted and corrected.

P10L281: Why use FEV1/FVC <0.70 instead of < LLM?

This is an important point. While LLN-based definitions provide greater physiological precision, we used the fixed ratio (FEV_1/FVC < 0.70) to maintain consistency with the original SHIP study protocols and to facilitate comparability across cohorts and with prior population-based and CPET reference studies. In this context, the fixed threshold represents a pragmatic approach aligned with large-scale epidemiological practice (e.g. Houtkamp et al., 2026).*

**Houtkamp D, Pool-Goudzwaard AL, Takken T, Chettouf S, Van de Wiel A, Bautmans I, Bongers BC. Reference values for cardiopulmonary exercise testing-derived parameters for cardiorespiratory fitness in Dutch community-dwelling 55- to 75-year-old adults. Eur J Appl Physiol. 2026 Feb;126(2):1067-1077. doi: 10.1007/s00421-025-05978-w)*

P14L385: The figure was reproduced with permission but I don't see the original citation.

The corresponding citation has been added to the figure legend and reference list.

P16L429: Should the authors include the actual constant they used here?

The constant has now been explicitly specified in the revised manuscript to improve clarity.

P16L433: I'm not sure it is entirely correct to say that achieving predicted MVV is "physiologically unattainable". Sometimes VEmax can exceed predicted MVV in athletes and clinical populations for a variety of reasons (e.g. exercise-induced bronchodilation, among other reasons).

We apologise for the imprecision in the original phrasing. The reviewer is correct in noting that predicted MVV can be approached or even exceeded under certain conditions, particularly in athletes. Our intention was not to suggest that MVV_pred itself is physiologically unattainable. Rather, the relevant constraint in the present framework is the combined condition defined by

MVV_pred × $FECO_2$_max, which represents a theoretical upper bound for ventilatory–gas exchange coupling. While high levels of ventilation may be achieved, the simultaneous attainment of maximal ventilatory capacity and maximal expired CO_2 fraction is highly unlikely under physiological conditions. This distinction has been clarified in the revised manuscript.

P19L506: Can the authors clarify why they ultimately decided on 41 as the constant for predicting MVV from FEV1?

We appreciate the reviewer's important question. The choice of 41 was made primarily for methodological consistency with the SHIP CPET framework from which the reference cohort was derived, as the original SHIP reference study calculated MVV as FEV_1 × 41. This multiplier is also retained in contemporary German CPET standard operating procedures, including the DZHK spiroergometry SOP, which explicitly defines calculated MVV as FEV_1 × 41 and cites both SHIP and Wasserman-based CPET methodology.

Table 1 is missing units for VO2. Also, RER should be reported to 2 decimal places.

Corrected.

Figure 7 is very informative.

We appreciate the reviewer's positive remark.

In general, figure resolution is quite poor. You can really notice this when you zoom in on the figures. I imagine it won't look great in final published form. The authors are encouraged to improve figure resolution.

We appreciate the reviewer for highlighting this point. All figures have been re-exported and reformatted in high resolution (publication-quality TIFF) in accordance with The Journal of Physiology guidelines. Resolution and clarity have been substantially improved in the present submission to ensure optimal visualization upon review.

We hope that these revisions have adequately addressed the reviewer's comments and improved the clarity and robustness of the manuscript.

Dear Dr Muller,

Re: JP-TFP-2026-290456R1 "**Ventilatory Efficiency: Physiological Modelling and Mechanistic Validation**" by PAULO de TARSO MÜLLER, BEATE STUBBE, TILL ITTERMANN, ERLANDSON FERREIRA SARAIVA, J. Alberto Neder, and RALF EWERT

Thank you for submitting your revised Technique to The Journal of Physiology. It has been assessed by the original Reviewing Editor and Referees and has been well received. Some final revisions have been requested.

Please advise your co-authors of this decision as soon as possible.

The referee reports are copied at the end of this email.

Please address all the points raised and incorporate all requested revisions or explain in your Response to Referees why a change has not been made. We hope you will find the comments helpful and that you will be able to return your revised manuscript within 4 weeks. If you require longer than this, please contact journal staff: jp@physoc.org.

Your revised manuscript should be submitted online using the link in your Author Tasks Link Not Available. This link is accessible via your account as Corresponding Author; it is not available to your co-authors. If this presents a problem, please contact journal staff (jp@physoc.org). Image files from the previous version are retained on the system. Please ensure you replace or remove any files that are being revised.

If you do not wish to submit a revised version of your manuscript, you must inform our journal staff (jp@physoc.org) or reply to this email to request withdrawal. Please note that a manuscript must be formally withdrawn from the peer review process at one journal before it may be submitted to another journal.

TRANSPARENT PEER REVIEW POLICY: To improve the transparency of its peer review process The Journal of Physiology publishes online, as supporting information, the peer review history of all articles accepted for publication. Readers will have access to decision letters, including Editors' comments and referee reports, for each version of the manuscript, as well as any author responses to peer review comments. Referees can decide whether or not they wish to be named on the peer review history document.

ABSTRACT FIGURES: Authors are expected to use The Journal's premium BioRender account to create/redraw their Abstract Figures. Information on how to access this account is here: https://physoc.onlinelibrary.wiley.com/journal/14697793/biorender-access.

This will enable Authors to create and download high-resolution figures. If authors have used the free BioRender service, they can use the instructions provided in the link above to download a high-resolution version suitable for publication.
The link provided should only be used for the purposes of this submission. Authors will be charged for figures created on this account if they are not related to this manuscript submission.

LANGUAGE EDITING AND SUPPORT FOR PUBLICATION: If you would like help with English language editing, or other article preparation support, Wiley Editing Services offers expert help, including English Language Editing, as well as translation, manuscript formatting, and figure formatting at www.wileyauthors.com/eeo/preparation. You can also find resources for Preparing Your Article for general guidance about writing and preparing your manuscript at www.wileyauthors.com/eeo/prepresources.

RIGOUR AND REPRODUCIBILITY: We recommend authors consult The Journal's Rigour and Reproducibility requirements to ensure that their manuscript is suitable for publication: https://physoc.onlinelibrary.wiley.com/pb-assets/hub-assets/physoc/documents/TJP-Rigour-and-Reproducibility-Requirements.pdf

REVISION CHECKLIST:
Check that your Methods section conforms to journal policy: https://jp.msubmit.net/cgi-bin/main.plex?form_type=display_requirements#methods

Check that data presented conforms to the statistics policy: https://jp.msubmit.net/cgi-bin/main.plex?form_type=display_requirements#statistics

Upload a full Response to Referees file. To create your 'Response to Referees' copy all the reports, including any comments from the Senior and Reviewing Editors, into a Microsoft Word, or similar, file and respond to each point, using font or background colour to distinguish comments and responses and upload as the required file type.

Please upload two versions of your manuscript text: one with all relevant changes highlighted and one clean version with no changes tracked. The manuscript file should include all tables and figure legends, but each figure/graph should be uploaded as separate, high-resolution files. The journal is now integrated with Wiley's Image Checking service. For further details, see: https://www.wiley.com/en-us/network/publishing/research-publishing/trending-stories/upholding-image-integrity-wileys-

image-screening-service

You may also upload:
- 'Potential Cover Art' for consideration as the issue's cover image
- Appropriate Supporting Information (Video, audio or data set: see https://jp.msubmit.net/cgi-bin/main.plex?form_type=display_requirements#supp).

We look forward to receiving your revised submission.

If you have any queries, please reply to this email and we will be pleased to advise.

Yours sincerely,

Paul Greenhaff
Senior Editor
The Journal of Physiology

REQUIRED ITEMS:

- You must start the Methods section with a paragraph headed Ethical Approval. If experiments were conducted on humans, confirmation that informed consent was obtained, preferably in writing, that the studies conformed to the standards set by the latest revision of the Declaration of Helsinki and that the procedures were approved by a properly constituted ethics committee, which should be named, must be included in the article file. If the research study was registered (clause 35 of the Declaration of Helsinki), the registration database should be indicated, otherwise the lack of registration should be noted as an exception (e.g. The study conformed to the standards set by the Declaration of Helsinki, except for registration in a database). For further information see: https://physoc.onlinelibrary.wiley.com/hub/human-experiments.

- A Data Availability Statement is required for all papers reporting original data. This must be in the Additional Information section of the manuscript itself. It must have the paragraph heading 'Data Availability Statement'. All data supporting the results in the paper must be either: in the paper itself; uploaded as Supporting Information for Online Publication; or archived in an appropriate public repository. The statement needs to describe the availability or the absence of shared data. Authors must include in their statement: a link to the repository they have used, or a statement that it is available as Supporting Information; reference the data in the appropriate sections(s) of their manuscript; and cite the data they have shared in the References section. Whenever possible, the scripts and other artefacts used to generate the analyses presented in the paper should also be publicly archived. If sharing data compromises ethical standards or legal requirements then authors are not expected to share it, but must note this in their statement. For more information, see our Statistics Policy.

EDITOR COMMENTS

Reviewing Editor:

Ethics Concerns:
None

Comments to the Author:
Thank you for this extensive revision. I was pleased to see that you addressed the issues I raised at the first review (that were not covered in the point by point rebuttal to the referees.)

My only remaining comment is about the graphical abstract. It appears to be more like poster for a scientific meeting that would provide some graphics for more context and discussion than can be presented in the abbreviated figure captions.I'm sorry I can't be more specific about how to simplify them but you can just look at those that pop up on the journal website for new publications to consider how you might simplify this part.

Senior Editor:

Thank you for the revised manuscript and rebuttal document, which have been considered by the same reviewing editor and reviewers that considered the original submission. I am happy to convey that all were of the opinion that the authors have done a good job at addressing the previous review comments and that the revised manuscript has been improved. The manuscript is now deemed potentially acceptable for publication following some minor modification requests. Firstly, please

indicate (or otherwise) in the Methods whether this large study represents a secondary, retrospective analysis of data. Secondly, whether the study was registered as a clinical trial (NOTE the Journal of Physiology guidance stipulates that is the research study was registered (clause 35 of the Declaration of Helsinki) the registration database should be indicated, else the lack of registration should be noted as an exception (e.g. The study conformed to the standards set by the Declaration of Helsinki, except for registration in a database.). The Reviewing Editor has requested that authors consider simplifying the graphical abstract so it 'stands alone' more, such that it is able to be understood better without reading the paper. Please note the graphical abstract is a good way to attract a reader to read the paper and is not meant to be a comprehensive presentation of findings. Thank you for supporting The Journal of Physiology. We look forward to receiving the further revised manuscript.

REFEREE COMMENTS

Referee #1:

I thank the authors for their careful consideration of my previous comments, and for the significant improvements introduced in the manuscript. Although I still think that the manuscript could be too long and complex for the average reader of the Journal, the authors propose a novel and interesting approach to the analysis of ventilatory efficiency during exercise in humans, which could significantly impact future work on the topic.

Referee #2:

The authors have done a good job addressing my comments.

END OF COMMENTS

Point-by-Point Response

JP-TFP-2026-290456R1 "Ventilatory Efficiency: Physiological Modelling and Mechanistic Validation"

Authors: Paulo de Tarso Müller, Beate Stubbe, Till Ittermann, Erlandson Ferreira Saraiva, J. Alberto Neder, and Ralf Ewert.

Dear Professor Greenhaff,
Dear Reviewing Editor,
Dear Referees,

We sincerely thank you for the careful re-evaluation of our revised manuscript and for the very encouraging assessment of this work. We were particularly encouraged to learn that both the Reviewing Editor and the Referees considered that the previous concerns had been adequately addressed and that the manuscript had improved. We also greatly appreciate the opportunity to perform these final refinements before publication.

We hope that these final modifications have fully addressed the remaining editorial requests.

EDITOR COMMENTS

Reviewing Editor

Comments to the Author: Thank you for this extensive revision. I was pleased to see that you addressed the issues I raised at the first review (that were not covered in the point by point rebuttal to the referees.)

My only remaining comment is about the graphical abstract. It appears to be more like poster for a scientific meeting that would provide some graphics for more context and discussion than can be presented in the abbreviated figure captions. I'm sorry I can't be more specific about how to simplify them but you can just look at those that pop up on the journal website for new publications to consider how you might simplify this part.

We sincerely thank the Reviewing Editor for this thoughtful and very helpful observation. In response, the graphical abstract has been comprehensively redesigned with the specific aim of improving stand-alone clarity, visual economy, and conceptual immediacy. Several secondary graphical elements were removed, text density was substantially reduced, explanatory content was simplified, and the visual narrative was reorganized to focus exclusively on the essential physiological sequence underlying the proposed framework—from conventional submaximal slopes, through semi-logarithmic transformation, to ceiling-normalized ventilatory efficiency ($\eta\dot{V}_E$*).*

We would also like to respectfully apologise to the Reviewing Editor for inadvertently not explicitly addressing some of his valuable comments and questions in our previous point-by-point response document, although the corresponding revisions had been incorporated into the revised manuscript. This oversight was entirely unintentional, and we are grateful for the opportunity to clarify these points explicitly in the present response.

Senior Editor

Thank you for the revised manuscript and rebuttal document, which have been considered by the same reviewing editor and reviewers that considered the original submission. I am happy to convey that all were of the opinion that the authors have done a good job at addressing the previous review comments and that the revised manuscript has been improved. The manuscript is now deemed potentially acceptable for publication following some minor modification requests. Firstly, please indicate (or otherwise) in the Methods whether this large study represents a secondary, retrospective analysis of data. Secondly, whether the study was registered as a clinical trial (NOTE the Journal of Physiology guidance stipulates that is the research study was registered (clause 35 of the Declaration of Helsinki) the registration database should be indicated, else the lack of registration should be noted as an exception (e.g. The study conformed to the standards set by the Declaration of Helsinki, except for registration in a database.). The Reviewing Editor has requested that authors consider simplifying the graphical abstract so it 'stands alone' more, such that it is able to be understood better without reading the paper. Please note the graphical abstract is a good way to attract a reader to read the paper and is not meant to be a comprehensive presentation of findings. Thank you for supporting The Journal of Physiology. We look forward to receiving the further revised manuscript

We sincerely thank the Senior Editor for this encouraging assessment and for this important methodological clarification. Accordingly, the Ethical Approval section at the beginning of the Methods has been revised to explicitly state that the present investigation represents a secondary, retrospective analysis of previously collected de-identified data (highlighted in yellow in the revised manuscript). This clarification now appears directly in the revised manuscript to ensure full methodological transparency and compliance with journal requirements.

We are very grateful for this recommendation and fully agree with the interpretation of the role of a graphical abstract. As described above, the graphical abstract has therefore been redesigned and simplified to function as a more immediate and self-contained visual summary of the central physiological concept proposed in this work. The revised version now emphasizes conceptual clarity, reduced visual density, and direct communication of the essential methodological innovation.

REFEREE COMMENTS

Referee #1

I thank the authors for their careful consideration of my previous comments, and for the significant improvements introduced in the manuscript. Although I still think that the manuscript could be too

long and complex for the average reader of the Journal, the authors propose a novel and interesting approach to the analysis of ventilatory efficiency during exercise in humans, which could significantly impact future work on the topic.

We are sincerely grateful to the reviewer for this thoughtful reassessment and for the constructive guidance provided throughout the review process. We particularly appreciate the recognition that the manuscript has meaningfully improved and that the proposed framework may contribute meaningfully to future work in exercise and respiratory physiology. The reviewer's comments played an essential role in improving the clarity, structure, and accessibility of the present work, and we sincerely thank them for their careful consideration.

Referee #2

The authors have done a good job addressing my comments.

We truly appreciate the reviewer's positive re-assessment. The constructive comments provided throughout the review process significantly strengthened both the methodological presentation and the clarity of the final manuscript.

We hope that these final revisions have fully addressed all remaining comments, and we respectfully submit the revised manuscript for final consideration.

With our sincere appreciation,

Paulo de Tarso Müller

(on behalf of all authors).

Dear Professor MÜLLER,

Re: JP-TFP-2026-290456R2 "**Ventilatory Efficiency: Physiological Modelling and Mechanistic Validation**" by PAULO de TARSO MÜLLER, BEATE STUBBE, TILL ITTERMANN, ERLANDSON FERREIRA SARAIVA, J. Alberto Neder, and RALF EWERT

We are pleased to tell you that your paper has been accepted for publication in The Journal of Physiology.

TRANSPARENT PEER REVIEW POLICY: To improve the transparency of its peer review process The Journal of Physiology publishes online, as supporting information, the peer review history of all articles accepted for publication. Readers will have access to decision letters, including Editors' comments and referee reports, for each version of the manuscript, as well as any author responses to peer review comments. Referees can decide whether or not they wish to be named on the peer review history document.

The last Word (or similar) version of the manuscript provided will be used by the Production Editor to prepare your proof. When this is ready you will receive an email containing a link to Wiley's Online Proofing System. The proof should be thoroughly checked and corrected as promptly as possible.

Authors should note that it is too late at this point to offer corrections prior to proofing. Major corrections at proof stage, such as changes to figures, will be referred to the Editors for approval before they can be incorporated. Only minor changes, such as to style and consistency, should be made at proof stage. Changes that need to be made after proof stage will usually require a formal correction notice.

All queries at proof stage should be sent to: TJP@wiley.com

Are you on Twitter? Once your paper is online, why not share your achievement with your followers? Please tag The Journal (@jphysiol) in any tweets and we will share your accepted paper with our 30,000 followers!

If you would like to receive our 'Research Roundup', a monthly newsletter highlighting the cutting-edge research published in The Physiological Society's family of journals (The Journal of Physiology, Experimental Physiology and Physiological Reports), please click this link, fill in your name and email address and select 'Research Roundup': https://www.physoc.org/journals-and-media/membernews/

Yours sincerely,

Paul Greenhaff
Senior Editor
The Journal of Physiology

P.S. - You can help your research get the attention it deserves! Check out Wiley's free Promotion Guide for best-practice recommendations for promoting your work at www.wileyauthors.com/eeo/guide. You can learn more about Wiley Editing Services which offers professional video, design, and writing services to create shareable video abstracts, infographics, conference posters, lay summaries, and research news stories for your research at www.wileyauthors.com/eeo/promotion.

• IMPORTANT NOTICE ABOUT OPEN ACCESS: To assist authors whose funding agencies mandate immediate public access to published research findings, The Journal of Physiology allows authors to pay an Open Access (OA) fee to have their papers made freely available immediately on publication.

The Corresponding Author will receive an email from Wiley with details on how to register or log in to Wiley Authors where you will be able to place an order.

You can check if your funder or institution has a Wiley Open Access Account here:
https://authors.wiley.com/author-resources/Journal-Authors/open-access/author-compliance-tool.html

----

EDITOR COMMENTS

Reviewing Editor:

Ethics Concerns:
None

Comments to the Author:
Thank you for this further revision. The graphical abstract is much better, I think. One has to read the paper to fully appreciate it but you've introduced the problem and summarized the approach nicely. It will be interesting to see if this novel method finds a place in clinical practice.

Senior Editor:

Comments to the Author:
Thank you for the revised submission and for addressing the final comments raised by the Reviewing Editor and ensuring the paper complies with The Journal of Physiology ethical guidelines. All concerned are happy with the revisions made by the authors and I am pleased to convey that the manuscript is deemed acceptable for publication. Congratulations and thank you for choosing to publish your research in The Journal of Physiology.
